# Ecological indicators and biological resources for hydrocarbon rhizoremediation in a protected area

**DOI:** 10.3389/fbioe.2024.1379947

**Published:** 2024-04-12

**Authors:** Alice Melzi, Sarah Zecchin, Stefano Gomarasca, Alessandro Abruzzese, Lucia Cavalca

**Affiliations:** ^1^ Dipartimento di Scienze per gli Alimenti, la Nutrizione e l'Ambiente (DeFENS), Università degli Studi di Milano, Milano, Italy; ^2^ Dipartimento di Scienze e Politiche Ambientali (ESP), Università degli Studi di Milano, Milano, Italy; ^3^ Dipartimento di Scienze Agrarie e Ambientali (DISAA), Università degli Studi di Milano, Milano, Italy

**Keywords:** PGPR, rhizodegradation, diatoms, *Ulnaria*, protected areas restoration, bioremediation, total petroleum hydrocarbons

## Abstract

Spillage from oil refineries, pipelines, and service stations consistently leads to soil, food and groundwater contamination. Bacterial-assisted phytoremediation is a non-invasive and sustainable solution to eliminate or decrease the concentration of xenobiotic contaminants in the environment. In the present study, a protected area interested by a fuel discharge was considered to assess a bioremediation intervention. From the spill point, a plume of contamination flowed South-West into the aquifer, eventually reaching a wetland area. Soils, groundwaters and plants belonging to the species *Scirpus sylvaticus* (L.) were sampled. In the majority of the soil samples, concentrations of total petroleum hydrocarbons, both C ≤ 12 and C > 12, exceeded legal limits set forth in Directive 2000/60/EC. The analysis of diatom populations, used as ecological indicators, evidenced morphology alterations and the presence of *Ulnaria ulna* and *Ulnaria biceps* species, previously detected in hydrocarbon-polluted waters. Tests for phytotoxicity and phytodegradation, carried out in soil mesocosms, planted with *Zea mays* and *Helianthus annuus*, demonstrated that both species significantly contributed to the removal of total petroleum hydrocarbons. Removal of C ≤ 12 and C > 12 petroleum hydrocarbons was in the range of 80%–82% for *Z. mays* and 71%–72% for *H. annuus*. Microbial communities inhabiting high organic carbon and vegetated soils were more active in hydrocarbon degradation than those inhabiting subsoils, as evidenced by soil slurry experiments. The abundance of functional genes encoding toluene-benzene monooxygenase (*tbmD*) and alkane hydroxylase (*alkB*), quantified in environmental samples, confirmed that the plant rhizosphere recruited a microbial community with higher biodegradation capacity. Bacterial strains isolated from the sampling site were able to grow on model hydrocarbons (hexane, hexadecane and *o-, m-, p*-xylene) as sole carbon and energy sources, indicating that a natural bio-attenuation process was on-going at the site. The bacterial strains isolated from rhizosphere soil, rhizoplane and endosphere showed plant growth promoting traits according to *in vitro* and *in vivo* tests on *Z. mays* and *Oryza sativa*, allowing to forecast a possible application of bacterial assisted rhizoremediation to recover the protected area.

## Introduction

Global environmental contamination by total petroleum hydrocarbons (TPH) represents a major ecological concern, with 2.5 million tons of TPH being released annually into the soil and groundwater (Zawierucha et al., 2022). The main sources of hydrocarbon contamination include oil and natural gas wells, oil refineries, pipelines, and service stations (Khan et al., 2018). Diatoms, whose natural structure is heavily affected by contaminants, are crucial and sensitive ecological indicators of both organic and inorganic compounds pollution (Noga et al., 2016). The European directive 2000/60/EC outlines primary environmental standards and legal limits for contaminant concentrations in water and soil, establishing a framework for intervention in case of environmental damage (EC, 2000; Dlgs, 2006). The remediation of contaminated sites (*ex* or *in situ*) allows restoration to the pristine state (Hussain et al., 2022). While physico-chemical remediation techniques have drawbacks like slow progress, high cost, production of secondary intermediates (Khalid et al., 2021), bioremediation overcomes these limitations, representing an eco-friendly alternative (Sayed et al., 2021). Bioremediation exploits living organisms, such as microorganisms, fungi and/or plants, to eliminate or reduce the concentration of environmental contaminants, and it consists in either natural bioremediation, biostimulation, or bioaugmentation (Zawierucha et al., 2022). Several plant species show potential for removing TPH through phytoadsorption, uptake, accumulation in the aboveground harvest, and phytovolatilization mechanisms (Ite and Ibok, 2019). In these processes, plant exudates, rich in organic acids and other signal molecules, play essential roles in enhancing hydrocarbon bioavailability and attracting degrading bacteria (Ite and Ibok, 2019). The microorganisms residing in the rhizosphere and responsible for TPH biodegradation utilize organic contaminants as source of both carbon and electron to generate energy for their growth. This process involves enzymes such as oxygenases in aerobic environments and thiolases in anaerobic environments. In anoxic environments such as aquifers, sediments, and submerged soils, anaerobic bacteria play a crucial role combining TPH degradation with the respiration of alternative electron acceptors such as SO_4_^2-^, NO_3_^−^, Fe^3+^ and HCO_3_^ˉ^ (Liu et al., 2019). Nevertheless, aerobic microbial degradation is faster in soils, involving oxygenase class of enzymes for the mineralization of petroleum hydrocarbons to biomass, carbon dioxide and water (Abdelhafeez et al., 2022; Zawierucha et al., 2022).

The association between plants and microorganisms represents a promising strategy for the remediation of TPH-polluted sites (Elijah, 2022). *In situ* microbial-assisted phytoremediation exploits the ability of bacteria to degrade TPH, while the plant either mitigates the toxic effects of the pollutant or removes contaminants from soil and water (Bhatt et al., 2022). Biostimulation or bioaugmentation of indigenous plants grown on contaminated sites with allo- or autochtonous rhizobacteria optimizes plants’ degradation potential, thereby increasing the efficiency of phytoremediation (Vacheron et al., 2013). Depending on the type of contaminant (*i.e.,* organic or inorganic), different phytoremediation mechanisms take place. Inorganic contaminants, such as heavy metals, can only be removed by extraction and transformation. In fact, several studies suggest using metal-resistant plants in association with rhizobacteria for phyto-stabilization purposes, aimed to limit the mobility of heavy metals (Bennis et al., 2022). On the other hand, organic contaminants such as hydrocarbons and chlorinated compounds can be removed by degradation, rhizoremediation, stabilization, and volatilization (Vocciante et al., 2022). Rhizosphere bacteria and endophytes that promote plant growth are classified as plant growth promoting rhizobacteria (PGPR) (Hoang et al., 2021a). Rhizosphere soils in petroleum-contaminated sites are often a rich source of microorganisms with the metabolic capability to degrade organic contaminants while promoting plant growth (Eze et al., 2022). PGPR provide plants with essential nutrients. As an example, some PGPR synthesize and secrete siderophores, low-molecular-weight molecules with a high affinity for Fe(III), which chelate the metal and facilitate its absorption by plant cells. Beside Fe, bacterial siderophores can chelate other metals, favoring their uptake by plants through phytoextraction (Bennis et al., 2022). PGPR can also modulate plant hormonal levels. In fact, almost 80% of bacterial strains inhabiting the rhizosphere produce auxin (*i.e*., indole-3-acetic acid, IAA) as a secondary metabolite (Deosthali and Sajwani, 2022). The application of bacterial-assisted rhizoremediation may be crucial to facilitate environmental recovery while preserving the local ecosystem of protected areas (El-Gendy, 2022). When agricultural activities are carried out within protected areas, it is important to select plants that are both useful for phytoremediation and easy to manage for farmers. Grass species belonging to the *Fabaceae* and *Poaceae* families have been reported as suitable candidates for TPH phytoremediation in hydrocarbon-impacted soils, while also contributing to ecosystem functioning (Yousaf et al., 2022).

The aim of this study was to evaluate the bioremediation potential of native bacterial communities and local plants present in a protected area affected by hydrocarbon contamination, to envisage the feasibility of implementing bacterial-assisted rhizoremediation for the reclamation of the protected site.

## Materials and methods

### Site description

A gasoline spill took place in a Site of Community importance (SCI) within the European Nature 2000 network of protected areas in the Po Valley (Lombardy, Italy). Springs and ditches consist of aquatic flora as *Robinia pseudoacacia* L., *Carex remota* L. and *Scirpus sylvaticus* L., and of a peculiar fish community. This includes many species of cyprinids, perch, bullhead (*Cottus gobio* Linnaeus, 1758), Italian pike (*Esox cisalpinus*, Bianco and Delmastro, 2011), and two rare endemic species, included in Habitat Directive, annex II, the Padana lamprey (*Lethenteron zanandreai* Vladykov, 1955) and the marble trout (*Salmo trutta marmoratus*, Cuvier, 1829). The latter two are classified as species in “critical danger” status, according to the International Union for Conservation of Nature (IUCN).

A gasoline pipeline, located 5 m below ground, was deliberately damaged during an attempted fuel theft, resulting in the contamination of an area encompassing 10,000 m^2^, including fields, groundwater, and ditches. The area between the oil spill point and the water collection channel has a slope, with a height of approximately 15 m, due to its position along a river paleochannel. Along the descent, some “terrace springs” are present. At first, physical remediation was applied using adsorbing materials barriers and pump-and-treat of contaminated waters through activated carbon filters. Since the physico-chemical treatments were not sustainable in the long-term, a bioremediation intervention was envisaged. The ecological impact of persistent contamination on the natural environment was assessed through the analysis of benthic diatom communities. The feasibility of a bacterial-assisted phytoremediation intervention was evaluated by determining the potential for natural attenuation of contaminants, conducting phytotoxicity tests, and characterizing plant growth promoting (PGP) and hydrocarbon-degrading bacteria.

### Diatom sampling and analysis

The ecological impact of ongoing contamination on the natural water environment was assessed by analyzing the benthic diatom communities (according with the Italian D. Lgs. 152/2006 and 260/2010 and European Legislation, 2000/60/EC) over a time frame of 3 years. Diatom communities are very sensitive to many types of pollutants such as nutrients, heavy metals, hydrocarbons, pesticides, and antibiotics. They have a rapid reproduction cycle that allows to detect short-term pollution phenomena effectively.

For diatom analyses, six white tiles were randomly placed in four stations, two contaminated springs, named ST2 and ST3 and two not-contaminated control stations along the ditch that collects water from different clean springs, named ST1 and ST4. Collected samples were stored separately, while a portion of each sample was pooled to form the “study sample”, representative of the typical population in ditches (ST1 and ST4) and in springs (ST2 and ST3). Samples were collected on hard substrates (rocks, mud, artificial substrates, sampling tiles) according to Mancini and Sollazzo (Mancini and Sollazzo, 2009) protocol, elaborated within the international standards indications (CEN EN 13946 and CEN EN 14407). Diatoms were harvested onto 30 cm-side white tiles. These were used to promote epiphytic diatom anchoring and photosynthesis. Samples were prepared for the analysis following the indications of the “Sampling and analysis protocol for benthic diatoms of Italian watercourses”, a APAT document (APAT and Parte, 2007). Briefly, samples were digested in 30% (vol/vol) hydrochloric acid for 30 min, washed in 50% (vol/vol) ethanol, and further digested in hydrogen peroxide (30 volumes) at 20°C for 4 days (APAT and Parte, 2007). Clean frustules were washed several times with 50% ethanol, mounted on glass slides with a drop of Naphrax, identified at species level and counted under a light microscope at 1,200x magnification (APAT-MATTM protocol).

Canonical correspondence analysis (CCA) was performed according to Dell’Acqua et al. (2014) to identify highly correlated linear combinations between diatom species distribution and environmental data information.

Data related to diatom population structures were processed using Past 4.03 (Hammer and Harper, 2024).

### Site sampling and physico-chemical characterization

Sediment samples of superficial waters were collected in stations ST1, ST2, ST3 and ST4 and analyzed for metal content by inductively coupled plasma–optical emission spectroscopy (ICP-OES) analysis (Analytical methods).

**FIGURE 1:**
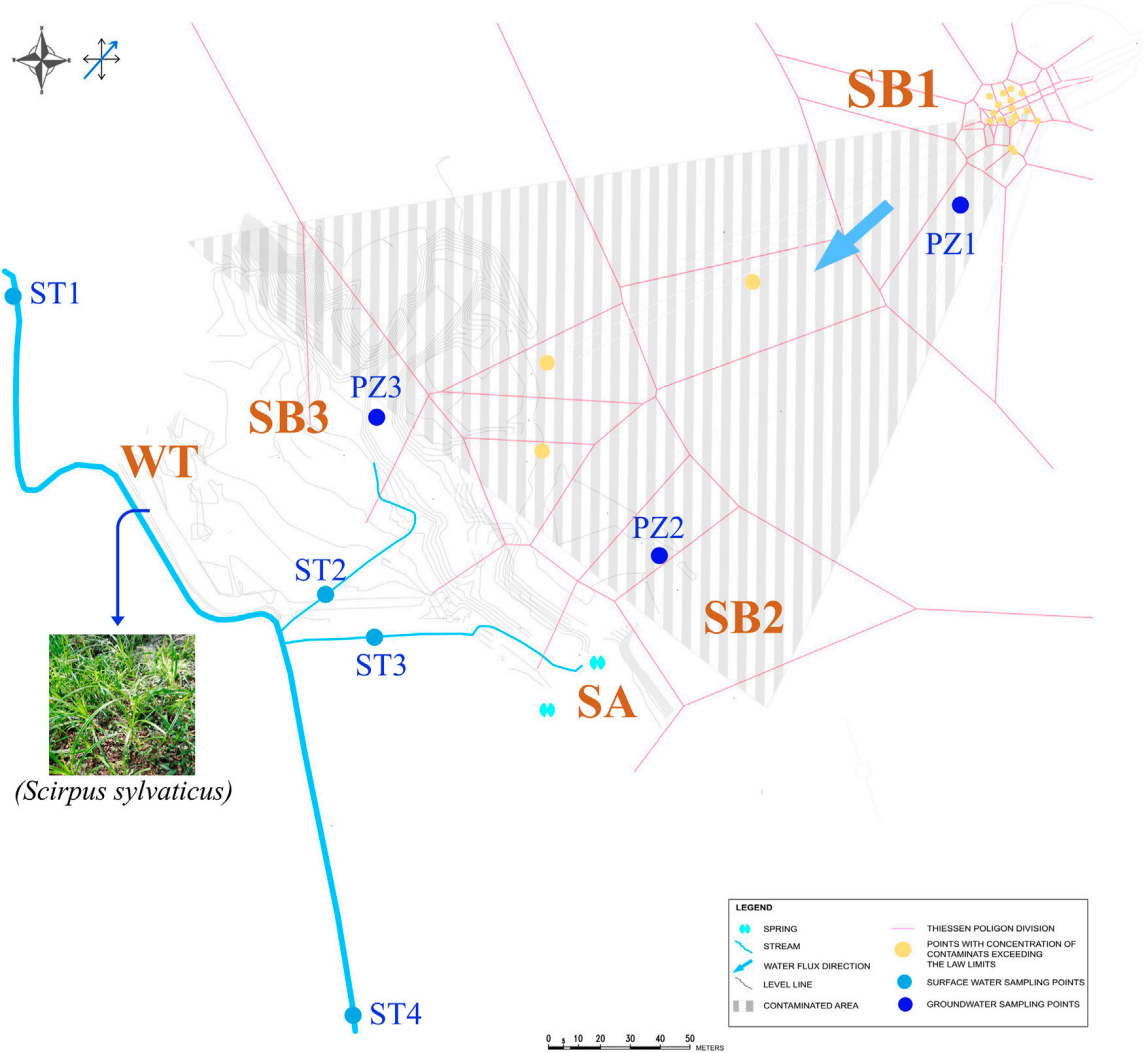
Spatial distribution of soil (SB1, SB2, SA, SB3 and WT), groundwater (PZ1, PZ2 and PZ3), sediment (ST1, ST2, ST3 and ST4) and plant (*Scirpus sylvaticus*) samples considered at the polluted site.

Soil samples were collected along the flow profile and terrain sloping profile (Figure 1) by drilling at a depth of 4.0–4.5 m below ground at the oil spill point (SB1) and within the contaminated plume (SB2), at a depth of 5 cm below ground (*i.e*., in the ‘O’ profile) at the shore of the spring (SA), and at a depth of 1.5 m depth in a grassland area (SB3). In the wetland area, bulk paddy soil was sampled at a depth of 30 cm below ground (WT). In the same wetland area, the dominant plant (*S. sylvaticus*, (L.)) was sampled and rhizosphere soil (WT-RS), rhizoplane (WT-RP) and root endosphere (WT-EN) were separated from the root system according to Cavalca et al. (2010), Luo et al. (2011), and Chen et al. (2012).

The samples were stored at 4°C in glass and plastic containers until microbiological and chemical analyses were performed, respectively.

Soil texture and pH were determined according to the official method n°II.5, n°III.1 (ISO 10390) (Metodi Ufficiali di Analisi Chimica del Suolo, 1999). Total organic carbon (TOC) and cation exchange capacity (CEC) were determined according to the official method of soil chemistry analysis VII.3 and XIII.2 (ISO 14235 and ISO 11260). Total nitrogen (TKN) was determined according to the Kjeldahl method (Kirk, 1950). Bioavailable phosphorus was determined according to either the Olsen or the Bray and Kurtz method, depending on soil pH (*i.e.,* pH > 6.5 or pH < 6.5, respectively) (Bray and Kurtz, 1945; Olsen et al., 1954). Metal content in soil samples was determined through inductively coupled plasma mass spectrometry (ICP-MS) and TPH by gas chromatography-mass spectrometry (GC-MS) analysis (Analytical Methods).

Groundwater was collected from piezometers PZ1, PZ2 and PZ3 between 0.3–1 m of the water column depth. Metal content in the samples was investigated through ICP-OES and spectrophotometric analyses and TPH by gas chromatography–mass spectrometry (GC-MS) analysis (Analytical Methods).

All the samples were collected and analyzed in triplicates (Supplementary Table S1).

### Phytotoxicity and TPH phytodegradation in soil mesocosms

Phytotoxicity tests were performed on monocotyledons and dicotyledons model plants *Z. mays* (L.) and *H. annuus* (L.) to evaluate the effect of soil pollution on plant growth. For both plant species, five replicate pots were filled with 300 g of SB3 and WT soil samples, chosen as representative of vegetated soils in the area. Pots filled with 300 g of universal soil were used as not-contaminated controls. Three and five seeds were placed in each pot for *Z. mays* and *H. annuus*, respectively. Preliminary results of germination trials indicated that the two species (C4 and C3, respectively) had a different biomass development. Therefore, a different number of seeds per pot and per plant was used to provide homogenous light irradiation and water availability to both species. Plants were grown in a glasshouse at 25/20°C day/night temperature, with 16-h light period, and a photosynthetic active radiation not lower than 250 E m^-2^. s^-1^. The plants were grown for 38 days maintaining a constant water content in the pot at 30% of field capacity. At the end of the growth period, the plants were sampled, and their biomass was measured.

The DUALEX SCIENTIFIC^+^™ (Force A) was used on apical, fully expanded, leaves to quantify leaf blade pigments, such as chlorophyll and flavonoids, as indicators of stress conditions induced by the growth on polluted soil.

After 38 days from sowing, soil and shoot samples were collected from the phytodegradation experiments for dry weight determination (105 °C until reaching constant weight) and TPH analysis (Analytical methods). C > 12 mass balance and shoot bioconcentration factor (*SCF*) were calculated according to the following formulae:

$$Mass\;balance\;C>12={T_{0}}_{soil}-\left({T_{f}}_{soil}+{T_{f}}_{shoot}\right)$$

$$SCF=\frac{\left[C>12_{shoot\;mg/kg}\right]}{\left[\;C>12_{soil\;mg/kg}\right]}$$

### Hydrocarbon biodegradation in microcosm soil slurries

Aerobic soil slurry experiments were set up with soil samples SB1, SB2, SA, and SB3, to evaluate the biodegradation of aromatic and aliphatic hydrocarbons by native microbial communities. Triplicate sieved soil samples (15 g each) were suspended in 35 mL M9 mineral medium (Cavalca et al., 2000) in glass serum bottles sealed with Teflon butyl rubber stoppers. Abiotic controls were set up with 15 g of sterilized soil. Soil sterilization was performed by three successive heat treatments in autoclave alternating with three incubations at 30°C. Slurry soils were incubated at 28°C under shaking for 45 days. Xylenes (as a mixture of *ortho-, meta*- and *para*) and C ≤ 12 and C > 12 TPH were analyzed by GC-MS (Analytical methods). Hydrocarbons biodegradation was expressed as the difference between initial and final concentrations.

Enumeration of hydrocarbon-degrading bacteria was performed using the most probable number (MPN) method. For each sample, 3 g of soil were resuspended in 27 mL of sodium pyrophosphate. Hexane (SIGMA, St. Louis, Missouri, United States), as representative of C ≤ 12, hexadecane (Merck, St. Louis, Missouri, United States), as representative of C > 12 and *o-m*- and *p-*xylene (Merck, St. Louis, Missouri, United States), as representative of aromatic hydrocarbons, were added separately (400 mg L^-1^) as sole carbon and energy source to M9 mineral medium (Cavalca et al., 2000) in Teflon rubber sealed in 120 mL serum bottles (1:9, w/v, soil/M9) inoculated with 1 mL of 1:10 successive dilutions of soil samples, for a total 10 mL final volume. After 15 days of incubation under shaking at 28°C, the samples were considered positive in the presence of growth turbidity.

### Analytical methods

Dissolved metals concentration in sediments and in groundwater samples was determined by ICP-OES. Sediment freeze-dried homogenized samples (0.20 g) were mineralized with concentrated high purity HNO_3_ (6 mL) and ultrapure water (2 mL). The solution was diluted with ultrapure water (50 mL) and analyzed in triplicate. Appropriate dilutions of groundwater samples were also analyzed.

Fe(II) speciation was determined according to the orthophenantroline method (Loeppert and Inskeep, 1996). Fe(III) was calculated by subtracting Fe(II) to total Fe. Soluble oxidized Mn(III) was measured according to the o-tolidine spectrophotometric method, which quantifies Mn(III), Mn(IV) and Mn(VII) (Morgan and Stumm, 1965). Mn(II) was calculated by subtracting Mn(III), Mn(IV) and Mn(VII) to total Mn.

Heavy metal content of 0.5 mm-sieved SB1, SB2, SA, SB3 and WT soil samples, was determined by ICP-MS. All samples were acidified by 2% (v/v) HNO_3_ and added with internal standards [Scandium (4 5Sc), Yttrium (8 9Y), Terbium (15 9Tb)] at concentrations ranging from 0 to 1 mg L^−1^ and prepared from a multi-standard solution (Agilent Technologies, United States).

To each average value, a coefficient of variation relating to a Relative sTandard Deviation (RTD%) was attributed. RTD% is equal to the standard deviation divided by the mean x 100.

C ≤ 12 and C > 12 TPH in groundwater and soil samples and shoots were extracted according to EPA methods 5030C-(SW-846) (U.S. EPA, 2003a) and 8015C-(SW-846) (U.S. EPA, 2003b). The extracted samples were analyzed by GC-MS according to 8260D-(SW-846) (U.S. EPA, 2006). Specifically, the latter method was used to determine the concentrations of nonhalogenated volatile organic compounds and semi volatile organic compounds. Samples sealed in glass vials by PTFE rubber stoppers were heat-treated and the purge-and-trap procedure for the analysis of volatile organic compounds was applied. The samples were injected in a 5975B Gas Chromatograph-Mass Spectrometer (Agilent Technologies, Santa Clara, CA, United States) equipped with D3792 PoraBOND Q column (25 m × 0.32 mm, 5.00 μm) (Agilent Technologies, Santa Clara, CA, United Statesa). Helium at 250 °C was the carrier gas. Samples were injected manually with a split ratio of 3:1 and a split flow of 7.5 mL. Oven temperature conditions were 40 °C for 2 min and then 10 °C/min to 260° for 7 min.

### Isolation and characterization of hydrocarbon-degrading/PGP bacterial strains

Ninety-three bacterial colonies were isolated from plate counts on Tryptic Soy Agar (TSA, OXOID, Basingstoke, UK) inoculated with ten-fold serially diluted SB1, SB2, SB3, SA, WT and WT-RS soil samples, and on R2A (VWR BDH Chemicals, Radnor, Pennsylvania, US) inoculated with the WT-EN root endosphere sample. The ability of isolated strains to use hexadecane as sole carbon and energy source was tested in M9 liquid media according to Cavalca *et al.* (Cavalca et al., 2000). Hydrocarbon adhesion to hexadecane was assessed by BATH test according to Abdulla *et al.* (Abdulla et al., 2014). Zinc (Zn) resistance was measured spectrophotometrically by growth turbidity (OD_600nm_) after 5 days of incubation in triplicate 24-well plates in Tris Mineral Medium (TMM) (Mergeay et al., 1985) added with Na-gluconate at 0.6% w/v (TMMG) and 32 mg L^-1^ of ZnSO_4_ (TMMG-Zn). PGP abilities were ascertained for all the strains. Dinitrogen fixation was assessed by testing the growth in liquid Brown medium (Thompson and Wildermuth, 1989). Inorganic phosphate solubilization was tested in a solid medium added with insoluble tricalcium phosphate (Ca_3_(PO_4_)_2_) and verified by the development of a clear zone around the colonies according to Goldstein *et al.* (Goldstein and Liu, 1987). Indole-3-acetic acid (IAA) production was determined after growth in Dworkin and Foster liquid mineral medium (Dworkin and Foster, 1958) by colorimetric method according to Glickmann and Dessaux (Glickmann and Dessaux, 1995), in the presence of Salkowski reagent (Gordon and Weber, 1951). Siderophore secretion was determined according to Alexander and Zuberer (Alexander and Zuberer, 1991) in agar plates with Tryptic Soy Agar (TSA) medium including Cromo Azurol S (CAS) dye (Sigma-Aldrich, Germany). Orange halos around the colonies indicate siderophore excretion. EPS producing strains were screened on solid Santaella medium (Santaella et al., 2008). The motility of the isolated strains was tested according to Shields and Cathcart (2011) (Kelly and Fulton, 1953) in solid Nutrient Broth (NB, Sigma-Aldrich, S. Luis, Missouri US) medium added with 0.005% (w/v) of triphenyltetrazolium chloride (TTC).

### Assessment of *in vivo* PGP abilities: growth pouches experiment

To determine *in vivo* the bacterial ability to promote plant growth, growth pouches experiments were set-up according to Dell’Amico et al. (2008). According to the PGP characteristics, hydrocarbon biodegradation and metal resistance, *Rhodococcus kronopolitis* strain SA-12, *Pseudomonas koreensis* strain MORI-15 and *Aeromonas media* strain MORI-53 were selected as promising inoculants. Prior to seed inoculation, the strains were incubated at 28°C under shaking for 24 h in 60 mL of DF liquid medium (Dworkin and Foster, 1958) added with 0.2 g L^-1^ tryptophane, in order to induce IAA production recognized as root elongation promoter (Deosthali and Sajwani, 2022). *Z*. *mays* and *Oryza sativa* were selected as model plants. Seeds were sterilized in NaClO at 1.5% (v/v) for 15 min, then washed three times in sterile water. Seeds in the presence and in the absence of inoculants (10^8^ cells mL^-1^) were placed into growth pouches (five replicates) imbibed with 12 mL CaSO_4_ and incubated for 15 days. At the end of the experiment, wet and dry weight seedling biomass (105°C until constant weight) was determined. Sterilization and bacterization were verified on TSA agar plates.

### Molecular methods

Environmental DNA (eDNA) of samples SB1, SB2, SA, SB3, WT, WT-RS and WT-RP was isolated by DNeasy Powersoil Kit (QIAGEN) and used as a template in Real Time quantitative polymerase chain reaction (qPCR) experiments. After different attempts, it was not possible to retrieve eDNA from the endosphere of roots of *S. sylvaticus* sampled at WT soil (WT-EN). Total bacterial 16S rRNA gene was quantified according to Zecchin *et al.* (Zecchin et al., 2017), using the DNA of *Alcaligenes* sp. strain Dhal-L (Cavalca et al., 2013) as standard. Toluene-benzene monooxygenases (*tbmD*) gene was quantified according to Hendrickx *et al.* (Hendrickx et al., 2006) and Fierer *et al.* (Fierer et al., 2005), using the cloned gene belonging to *Pseudomonas veronii* as standard (Bertolini, 2021). Alkane hydroxylase (*alkB*) gene was quantified as in Kloos *et al.* (Kloos et al., 2006), using the DNA of *A. media* strain MORI-53 (present work) as standard. The number of gene copies in each sample was calculated from a calibration line obtained on standard curves with respective reference DNAs and threshold lines (Ct). Reaction efficiencies (%) were 136, 128 and 127 for 16S rRNA, *tbmD* and *alkB,* respectively. *R* ^2^ values were 0.98, 0.97 and 0.97 for the three genes, respectively. The DNA of the bacterial isolates was extracted by UltraClean Microbial DNA Isolation Kit (MO BIO). Isolated strain collection was de-replicated by Internal Transcribed Spacer (ITS) analysis according to Cardinale *et al.* (Cardinale et al., 2004). After de-replication, representative strains were identified by 16S rRNA gene nucleotide sequence (Microsynth, Switzerland) analysis.

## Results and discussions

### Physicochemical characterization of soil samples

Soil physico-chemical characteristics were measured to ensure the absence of factors that might hinder microbial activity and plant growth.

Ionomics revealed higher concentrations of arsenic, iron, and manganese in the contaminated sediments (ST2 and ST3) when compared to the control sites (ST1 and ST4, Supplementary Table S2). According to ICP-MS analysis, Zn was the only heavy metal exceeding law limits (D. Lgs. 152/2006 and Directive 2000/60/EC) in the bulk paddy soil sample WT of the wetland area, with 158 mg kg^-1^ d. w. (Supplementary Table S2). In this sample, total iron and aluminum were higher compared to the other samples, probably due to the reducing conditions of saturated soils that enhance metal dissolution. In groundwater samples, the concentration of heavy metals was under the law limits (D. Lgs. 152/2006 and Directive 2000/60/EC) (Supplementary Table S3). Soil samples were sub-acid/neutral and characterized by different sandy textures (Supplementary Table S4). TOC, TNK and CEC were higher in vegetated soil samples SA, SB3 and WT compared to subsoils SB1 and SB2, possibly due to the presence of higher organic matter within the organic “O” horizon.

TPH C ≤ 12 exceeded the law limits (D. Lgs. 152/2006 and Directive 2000/60/EC) at the oil spill point SB1, with 228 mg kg^-1^ d. w., and at the shore of the spring SA in the contaminated plume with 174 mg kg^-1^ d. w. TPH C > 12 exceeded law limits (D. Lgs. 152/2006 and Directive 2000/60/EC) in all samples, being sample SA the most affected one with 20,459 mg kg^-1^ d. w. (Table 1). This characterization indicated that TPH were transported from the oil spill point SB1 through the groundwater flow, down to the slope till the shore of the spring SA. Here, the soil, characterized by a high organic content, accumulated TPH C > 12 exceeding the limit by 400 times, potentially due to the retention of TPH by organic matter (Table 1; Supplementary Table S4). Groundwater samples were not affected by organic contaminants.

**TABLE 1:** Total petroleum hydrocarbon concentration in soil samples.

Samples	SB1	SB2	SA	SB3	WT	Law limits (directive 2000/60/EC)
C ≤ 12 (mg kg^-1^soil d.w.)	**228** ± 67	7 ± 1	174 ± 15	24 ± 6	22 ± 5	50
C > 12 (mg kg^-1^soil d.w.)	963 ± 80	41 ± 0.7	**20,459** ± 297	**206** ± 7	**222** ± 10	10

### Diatom population as ecological indicator of pollution

The species identified over three year-sampling were 160 and the total number of counted frustules was 8,101 (Supplementary Table S5A). The statistical analysis included 60 taxa, accounting for 95.57% of the total frustule count. The remaining 101 species, which overall represented 4.47% of the community, were not considered in the statistical analysis being little representative of the population structure. During the annual sampling period, starting from spring until the end of summer, an accumulation of black or orange-red encrusting material was observed on the surface of the white tiles (going from white to black or to orange-red). The red and black substrates were highly reactive to hydrogen peroxide used to digest the diatom and the substrate samples, probably due to an average presence of 1,266 mg L^-1^ Fe(II) and 419 mg L^-1^ Mn(II) over the sampling time. Manganese and iron are in fact two powerful catalysts that mediate the breaking reaction of hydrogen peroxide molecule with the release of oxygen.

**FIGURE 2:**
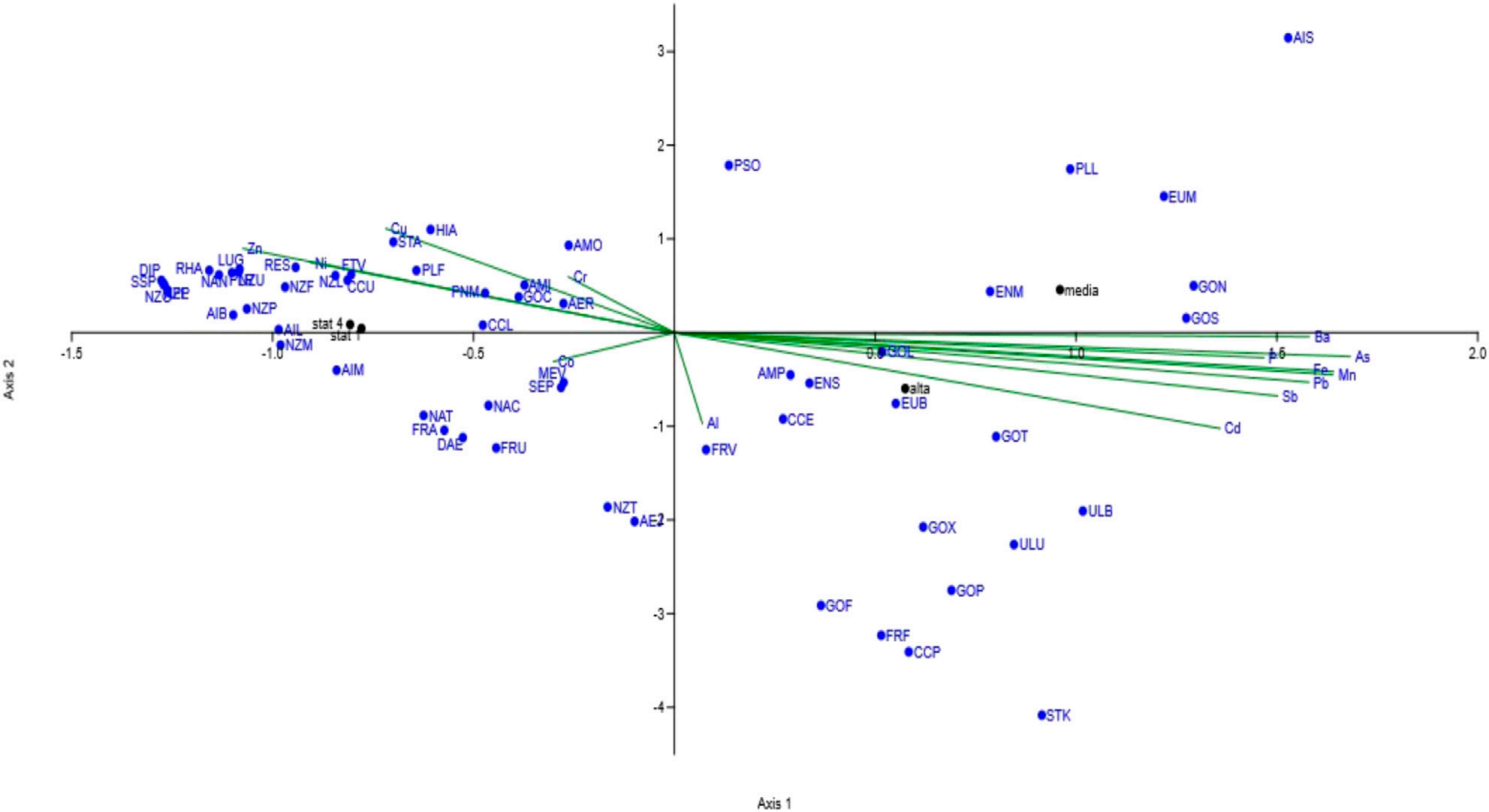
Canonical correspondence analysis performed on diatom populations and metal content of sediments.

The influence of metals on the structure of the diatom communities was evaluated through CCA (Figure 2). On axis 2, a distinct separation was evident between the metal pollution factors (environmental parameters) and the populations characterizing the control sites (ST1 and ST4). A different trend was also observed between ST2 and ST3 on axis 1.

Twenty-two different diatom species were exclusively present in the polluted sources (ST2 and ST3) (Supplementary Table S5B). These species were associated to high concentrations of iron, manganese and arsenic, to variable concentrations of aluminum, and to generally lower amounts of barium, lead, antimony and cadmium in the four sampling stations (Supplementary Table S2).

Thirty-eight species, exclusively present in ST1 and ST4 (Supplementary Table S5B), appeared to be linked to a relatively low and constant concentration of chromium, copper, zinc, cobalt (Supplementary Table S2).

In the two polluted stations (ST2 and ST3), the most represented diatom species (from 200 to over 3,300 frustules) were *Eunotia minor, Gomphonema angustatum, G. parvulum, Planothidium lanceolatum, Ulnaria biceps* and *Ulnaria ulna* (Supplementary Table S5B).

**FIGURE 3:**
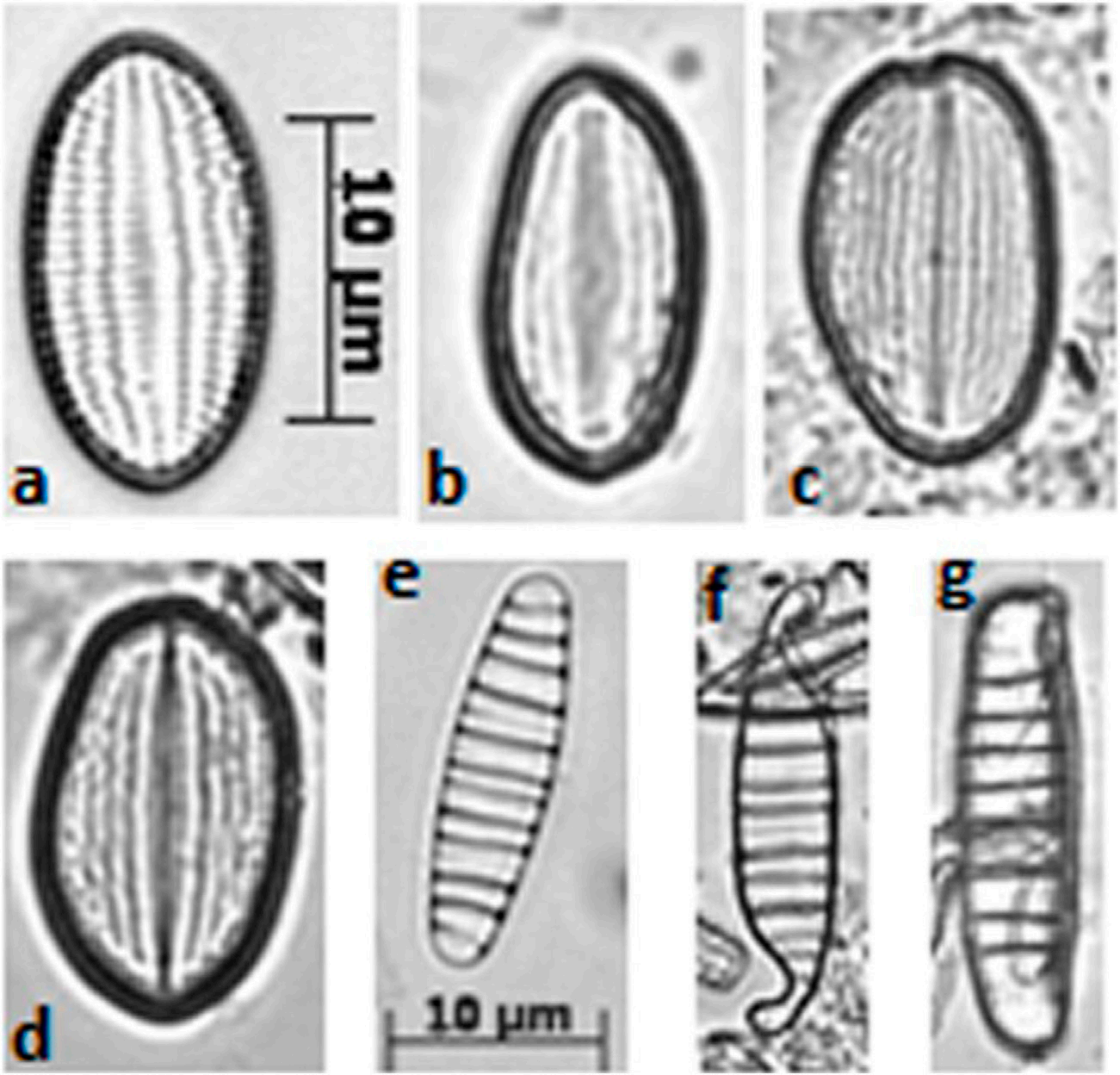
Morphological alteration of diatom frustules (optical microscope photographs, 1,200x). *Cocconeis pseudolineata* [from **(A–D)**] and *Diatoma moniliformis* [from **(E–G)**)]; **(A, E)** are normal shapes, while **(B, C, D, F, G)** are altered shapes.

Malformed frustules were found in ST2 and ST3, particularly within the genera *Cocconeis*, *Diatoma, Achnanthes/Achnanthidium* and *Planothidium* (Figure 3). In polluted sediments ST2 and ST3, indicator species (*G. angustatum, G. parvulum, P. lanceolatum, U. biceps* and *U. ulna*)*,* were prevalent in β mesotrophic and polysaprobic waters, while *E. minor* is more linked to α mesotrophic but basically acidic waters (Noga et al., 2016). The presence of *U. ulna* and *U. biceps* in sample ST3 is notable, since *U. uln*a is recognized as highly resistant and tolerant to water pollution (Celekli and Öztürk, 2014; Dalu et al., 2015; Noga et al., 2016). On the other hand, *U. biceps* was retrieved in waters with high electrical conductivity (Celekli and ÖZPINAR, 2021) and phosphate concentration (Celekli and Öztürk, 2014). A series of experiments on diatom sensitivity to herbicides showed that *U. ulna* and *U. biceps* are resistant to high atrazine concentrations (Chakandinakira et al., 2019). Furthermore, the two mentioned species were previously found in waters polluted by hydrocarbons (Henry and Charles, 2014).

*Amphora pediculus, Cocconeis euglypta, C. pediculus, Encyonema silesiacum, Eunotia bilunaris, Fragilaria capucina* var. *vaucheriae, Gomphonema parvulum* var. *parvulum f. saprophilum, G. clavatum, G. exilissimum* and *G. subclavatum* were present with 30–200 frustules (Supplementary Table S5A). The control stations (ST1 and ST4) were characterized by different diatom species, among which the most represented (from 200 to over 3,000 frustules) were *Achnanthidium biasolettianum, A. lineare, A. minutissimum, Cocconeis lineata, Navicula cryptocephala, Planothidium frequentssimum, Pseudomonas rostratum* (Supplementary Table S5B).

*Achnanthes minutissima* var. *jackii, A. rupestoides, Amphora inariensis, A. pediculus, Fragilaria ulna* var. *acus, Melosira varians, Nitzschia fonticola, Nitzschia palea, Reimeria sinuata, Rhoicosphenia abbreviate* and *Sellaphora pupula* were less frequent (from 30 to 200 frustules), but always characterized by a significant presence (Supplementary Table S5B).

When a contamination event takes place, ecological indicators such as diatoms are fundamental to evidence the impact on the whole biome. In the present study, the oil spill significantly affected the diatom community, evidencing that in this protected area a bioremediation intervention is strongly recommended to preserve/reestablish the naturalistic, agricultural or recreational vocation of the site (Yousaf et al., 2022).

### Soil phytotoxicity tests on agricultural plants and TPH removal

To assure agroecosystem functioning, the assessment of contaminant phytotoxicity and biodegradation potentials of plants-microbiome associations is essential. The plants of *Z. mays* and *H. annuus* grown for 38 days on SB3 and WT showed an evident reduction in growth. The aerial biomass was approximately 50% lower for *H. annuus* and 30%–40% for *Z. mays* compared to control plants, with the SB3 soil leading to the stronger effect, reducing biomass by 22%. Both polluted soils SB3 and WT led to an increase in root biomass. In particular, *H. annuus* exhibited an 80% increase in biomass, while *Z. mays* also showed a similar increase in root biomass, but only in the case of the WT soil.

**FIGURE 4:**
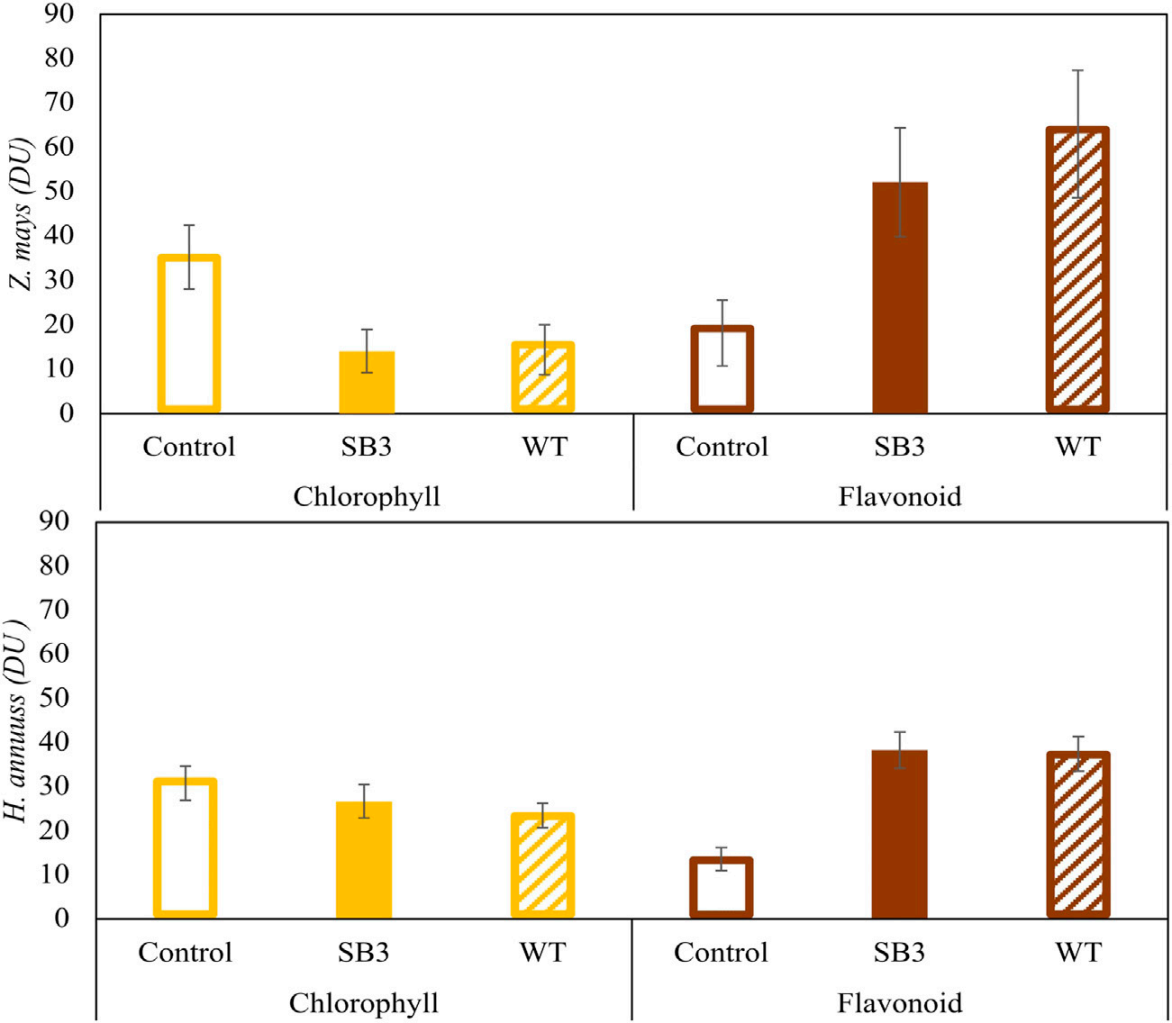
Chlorophyll and flavonoid contents in *Zea mays* and *Helianthus annus* grown in SB3 and WT soil mesocosms after 38 days.

Plant stress caused by growth on SB3 and WT soils was confirmed by the measurements of chlorophyll and flavonoids contents performed with the DUALEX device (Figure 4). When grown on SB3 and WT, *Z. mays* leaves showed a progressive reduction of chlorophyll, to a final content of 15 DU (Dualex Units) in both sides of the leaf blade. On the other hand, DU in the control plants was 30. Growth in both contaminated soils induced an increase of leave flavonoids in *Z. mays*, with content levels two to five times higher than those of control plants. *Helianthus annuus* exhibited a similar increase in flavonoids content but did not show a reduction in chlorophyll content.

For both *Z. mays* and *H. annuus*, the present data indicate a negative effect of soil contamination on plant growth, manifested by pronounced stress symptoms that affect plant biomass and the metabolism involved in pigments synthesis. According to the phytotoxicity tests, the transition from chlorophyll synthesis to flavonoids synthesis can be considered as a response to stress in the plants that induces a reduction in chlorophyll synthesis by altering nitrogen metabolism, consistent with findings from previous studies (Cerovic et al., 2012; Vishnupradeep et al., 2022).

After 38 days of plant growth, GC-MS analysis evidenced that C ≤ 12 and C > 12 TPH concentrations in soils were lower compared to the T_0_ (Table 2). C > 12 TPH were 75 and 30 mg kg^-1^ in grassland soil SB3 planted with *Helianthus annus* and *Z. mays,* respectively. These compounds were 60 and 55 mg kg^-1^ in soil WT planted with *H. annus* and *Z. mays*, respectively. Plants decreased soil hydrocarbon concentrations, with greater efficiency for C > 12 with respect to C *≤* 12 compounds. This was probably due to the presence of a higher concentration of C > 12 hydrocarbons than C *≤* 12. *Z. mays* was more efficient in decreasing C > 12 compounds in both soils compared to *H. annuus*. According to the phytodegradation tests performed in the present study, *Z. mays* was the best performing plant species in the removal of C ≤ 12 and C > 12 TPH. This confirms previous observations that *Z. mays* can be successfully used as a model plant, thanks to its rapid growth and high susceptibility to contamination (Cerovic et al., 2012). This plant species has been reported as capable of phytoextraction of pollutants from contaminated soils by roots-to-shoots translocation (Pacwa-Płociniczak et al., 2023).

**TABLE 2:** Total petroleum hydrocarbon concentration in planted (*Zea mays* and *Helianthus annus*) SB3 and WT soils, before (T_0_) and after 38 days (T_f_).

	C ≤ 12 (mg kg^-1^soil d.w.)			C > 12 (mg kg^-1^ soil d.w.)		
	T_0_	T_f_	Removal %	T_0_	T_f_	Removal %
SB3-*Helianthus annus*	73 ± 12	21 ± 4.5	71	150 ± 12	75 ± 12	50
SB3-*Zea mays*		13 ± 3.2	82		30 ± 11	80
WT- *Helianthus annus*	22 ± 4.5	12 ± 1.1	45	222 ± 32	60 ± 23	72
WT-*Zea mays*		17 ± 2.1	23		55 ± 17	75

C > 12 mass balance evidenced that hydrocarbons were volatilized or degraded in both soils and plants (Table 3), suggesting that contaminants were not accumulated in the plant shoots. Instead, they were probably volatilized or degraded by the indigenous microbial community which displayed this ability in microcosms experiments (see below). This result could indicate that the process is taking place at the site, where the wild vegetation is able to grow despite soil contamination. This was also confirmed by the bioconcentration factor (Supplementary Figure S1) used to determine the translocation of contaminants in plants. In fact, both plant species cultivated on WT soil absorbed and translocated less hydrocarbons C > 12 than the species grown on SB3 soil. This aspect may be related to the presence in WT of a higher content of organic matter that complexed pollutants, thereby reducing their bioavailability. Hence, the lower concentration of organic matter in SB3 soil could explain the higher plant uptake/translocation of contaminants. On the other hand, the presence of plants and roots in the soil promoted the recruitment of microorganisms with degrading capacities, emphasizing a rhizosphere effect in the degradation of organic contaminants. This effect has been studied by Panchenko *et al.* (Panchenko et al., 2022), who investigated the TPH content in rhizosphere and non-rhizosphere soil in a number of plant species.

**TABLE 3:** Mass balance of C > 12 hydrocarbons in *Helianthus annus* and *Zea mays* in SB3 and WT soil mesocosm, before (T_0_) and after 38 days (T_f_).

	C > 12 T_0_ (mg)	C > 12 T_F_ soil (mg)	C > 12 T_F_ shoot (mg)	C > 12 volatilized or degraded (mg)
SB3-*Helianthus annus*	124 ± 15.3	55 ± 6.23	0.05 ± 0.002	69 ± 4.23
SB3-*Zea mays*		16 ± 3.4	0.02 ± 0.003	108 ± 13.4
WT-*Helianthus annus*	177 ± 32	49 ± 5.34	0.02 ± 0.001	128 ± 16
WT-*Zea mays*		44 ± 5.34	0.04 ± 0.002	133 ± 22

### Slurry soils hydrocarbon biodegradation

**FIGURE 5:**
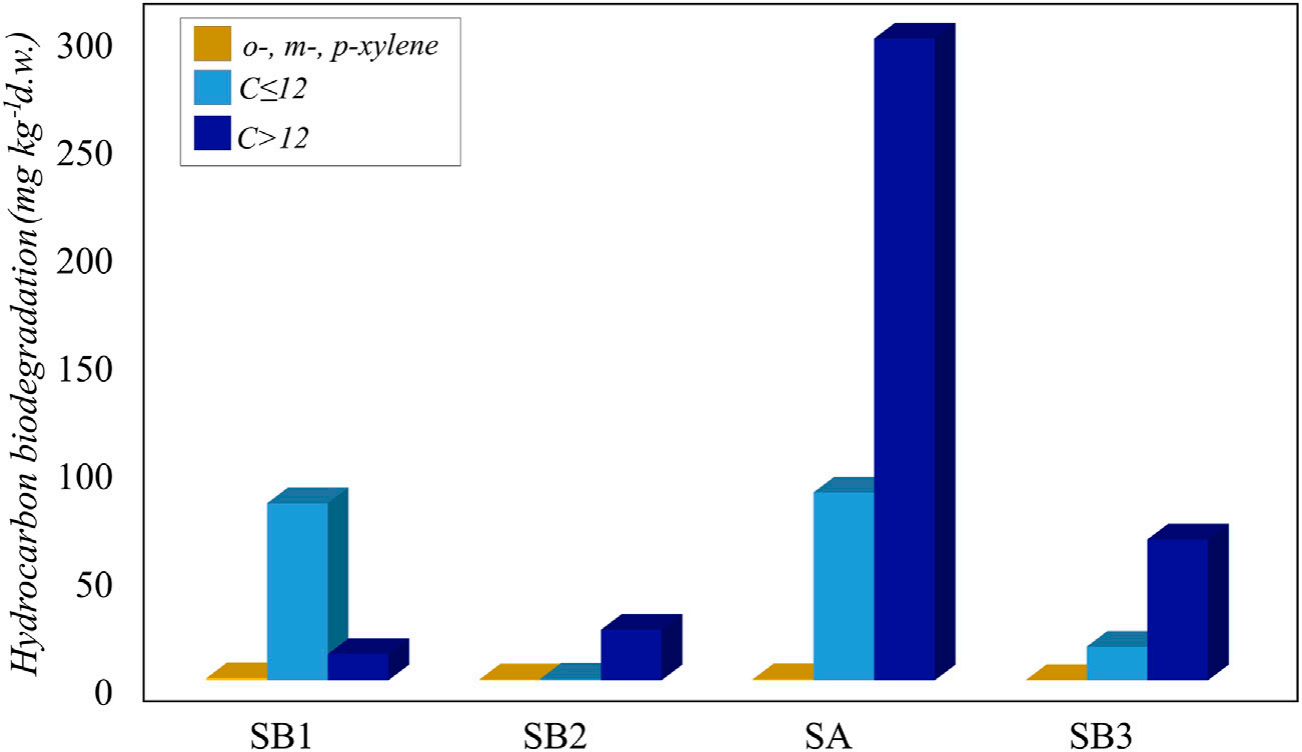
Biodegradation of hydrocarbons (*o-*, *m*- and *p*-xylene, C ≤ 12 and C > 12) in slurry soil microcosms.

Incubation for 45 days showed a higher degradation in the not sterilized soil slurries compared to the abiotic controls (Figure 5). For *o*-, *m*- and *p*-xylene and C ≤ 12, the abiotic physico-chemical degradation was irrelevant compared to biological degradation (Supplementary Table S6).

The ability to degrade light and heavy hydrocarbons was widespread in all the sampling sites. In particular, the most contaminated sample SA was also the most active in the degradation of C ≤ 12 and C > 12 petroleum hydrocarbons fractions, likely due to the presence of hydrocarbon-degrading bacteria. Although sample SB1 was collected at the spill point, C > 12 petroleum hydrocarbon degradation at this point was the lowest, possibly due to the surrounding environmental conditions, such as the absence of vegetation that promoted the recruitment of degrading bacteria (Figure 5).

**FIGURE 6:**
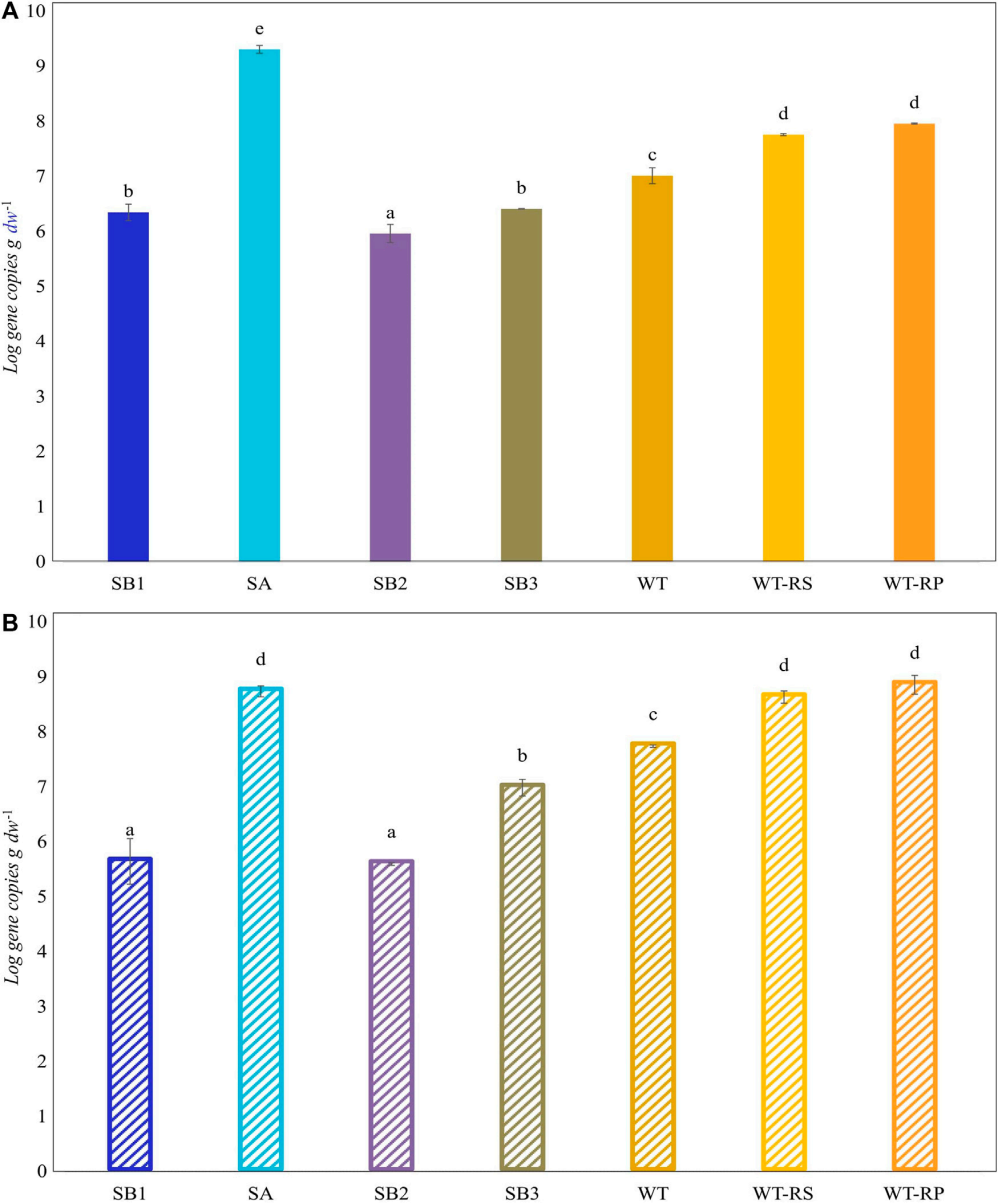
Abundance (Log scale) of *tbmD* **(A)** and *alkB* **(B)** biomarkers for toluene-benzene monooxygenase and alkane hydroxylase. Lowercase letters indicate significant differences between samples (Tukey’s test, *p* ≤ 0.05).

Functional biomarkers *tbmD* and *alkB* for toluene-benzene and alkane monooxygenases, respectively, were quantified by qPCR in eDNA isolated from soil samples SB1, SB2, SA, SB3, WT, and from rhizosphere soil WT-RS and rhizoplane WT-RP of one of the prevalent plant *S. sylvaticus* (Figure 6). *AlkB* gene copies abundance (10^8^ gene copies g^-1^ d. w.) was higher compared to *tbmD* gene (10^7^ gene copies g^-1^ d. w.) in almost all samples*. AlkB* genes, being highly widespread in nature, are frequently used as biomarkers to monitor and estimate petroleum and alkane bacterial degradation. Since alkanes are more easily degraded than aromatic compounds, we expected that *alkB* gene could have been higher than *tbmD*. Beside this, the difference between the two biomarkers is only of one order of magnitude and could also be attributed to experimental variation in PCR amplification/primer specificity (Paisse et al., 2011). The vegetated soils SA, SB3, WT, WT-RS and WT-RP were characterized by higher *tbmD* and *alkB* gene copies. Organic acids and root exudates, which may contain signal molecules such as flavonoids, can enhance the recruitment of hydrocarbon-degrading bacteria and plant colonization. This was confirmed by the higher number of viable bacteria in vegetated soils (10^7^–10^8^ CFU g^-1^ d. w.) compared to subsoils (10^6^ CFU g^-1^ d. w.). The same tendency was observed in viable hydrocarbon-degrading bacteria (Table 4). In vegetated soil samples, bacteria able to grow on C6 and C16 alkanes were two to four orders of magnitude higher than those in subsoils ones. Bacteria able to grow on *o-, m-* and *p-*xylene were less represented, and they were retrieved in subsoils without vegetation (SB1, SB2) and in one of the vegetated (SB3) samples.

**TABLE 4:** Quantification of hexane, hexadecane and xylenes degrading bacteria in subsoils (SB1, SB2) and vegetated (SA, SB3 and WT) soils.

	Degrading bacteria (MPN g^-1^ d.w.)		
Sample	Hexane 400 mg L^−1^	Hexadecane 400 mg L^−1^	Xylenes^a^ 400 mg L^−1^
SB1	2.5 × 10^3^ ± 0.27	3.4 × 10^5^ ± 0.5	4.8 × 10^1^ ± 0.18
SB2	2.01 × 10^4^ ± 0.11	4.9 × 10^3^ ± 0.63	7.53 × 10^3^ ± 0.4
SA	1.5 × 10^5^ ± 0.28	4.2 × 10^6^ ± 0.42	0
SB3	4.7 × 10^4^ ± 0.15	1.1 × 10^7^ ± 0.21	2.6 × 10^1^ ± 0.25
WT	1.5 × 10^4^ ± 0.22	4.78 × 10^7^ ± 0.11	0

^a^ equimolar mixture of *o*-, *m*- and *p*-xylene.

As a confirmation of a rhizosphere effect, the slurry soil microcosm experiment revealed that vegetated samples performed a better degradation of C ≤ 12 and C > 12. The presence in these samples of a higher number of viable hydrocarbon-degrading bacteria compared to the subsoils suggests the presence of an active hydrocarbon degradation *in situ*. This is in accordance with previous findings by Hoang *et al.* (Hoang et al., 2021b), who observed that in plant rhizosphere the type and quantity of contaminants present in the soil promote the enrichment of bacteria with degrading capacities.

The presence of the plant and its microbiota allows the decrease of pollutant content due to the higher prevalence, in the rhizosphere soil, of genes encoding enzymes involved in hydrocarbon degradation. Functional genes involved in hydrocarbon degradation (*tbmD* and *alkB*) were detected at the site and they significantly increased when at the proximity of the roots. Previous study (Ite and Ibok, 2019) evidenced that the recruitment of bacteria with catabolic activities increased while getting closer to the roots where organic acids secretion was higher.

### Characterization of PGP/hydrocarbon degrading bacterial strains

**FIGURE 7:**
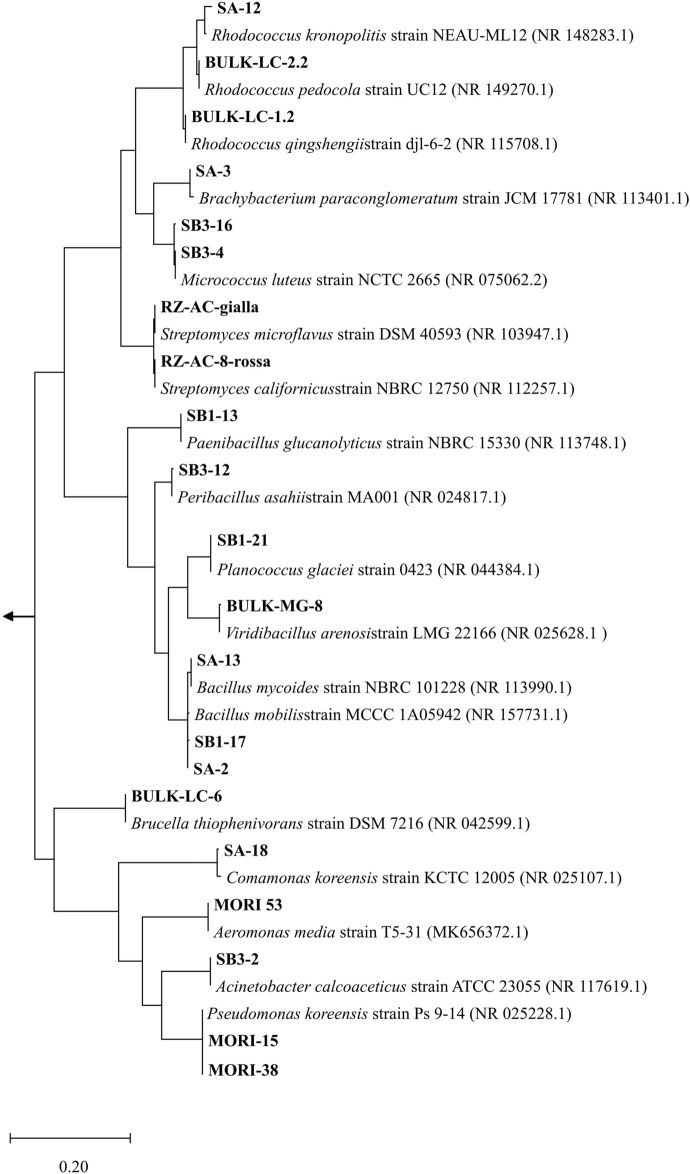
Phylogenetic affiliation (MEGA11) (Tamura et al., 2021) of the 21 OTUs according to 16S rRNA nucleotide sequence. The evolutionary history was inferred with the Maximum Likelihood method based on the Tamura–Nei model (Tamura and Nei, 1993). The tree is drawn to scale, with branch lengths measured in the number of substitutions per site. *Gloeocapsa* sp. (MW449597), *Calothrix* sp. (MN733320), *Leptolyngbya* sp. (MN733313) were used as outgroups.

In view of a bacterial-assisted bio/phyto-remediation intervention at the site, a total of 33 strains were isolated from soil samples (SB1, SB2, SA, SB3, WT, WT-RS) and 60 strains from root endosphere (WT-EN) of *S. Sylvaticus* (Supplementary Table S7). After dereplication by ITS profiling, the strain collection was organized in 21 OTUs. According to 16S rRNA nucleotide sequence identification, the most abundant strains belonged to the genera *Rhodococcus* (3 strains), *Bacillus* (3 strains), *Micrococcus luteus* (2 strains) and *P. koreensis* (2 strains) (Figure 7).

**FIGURE 8:**
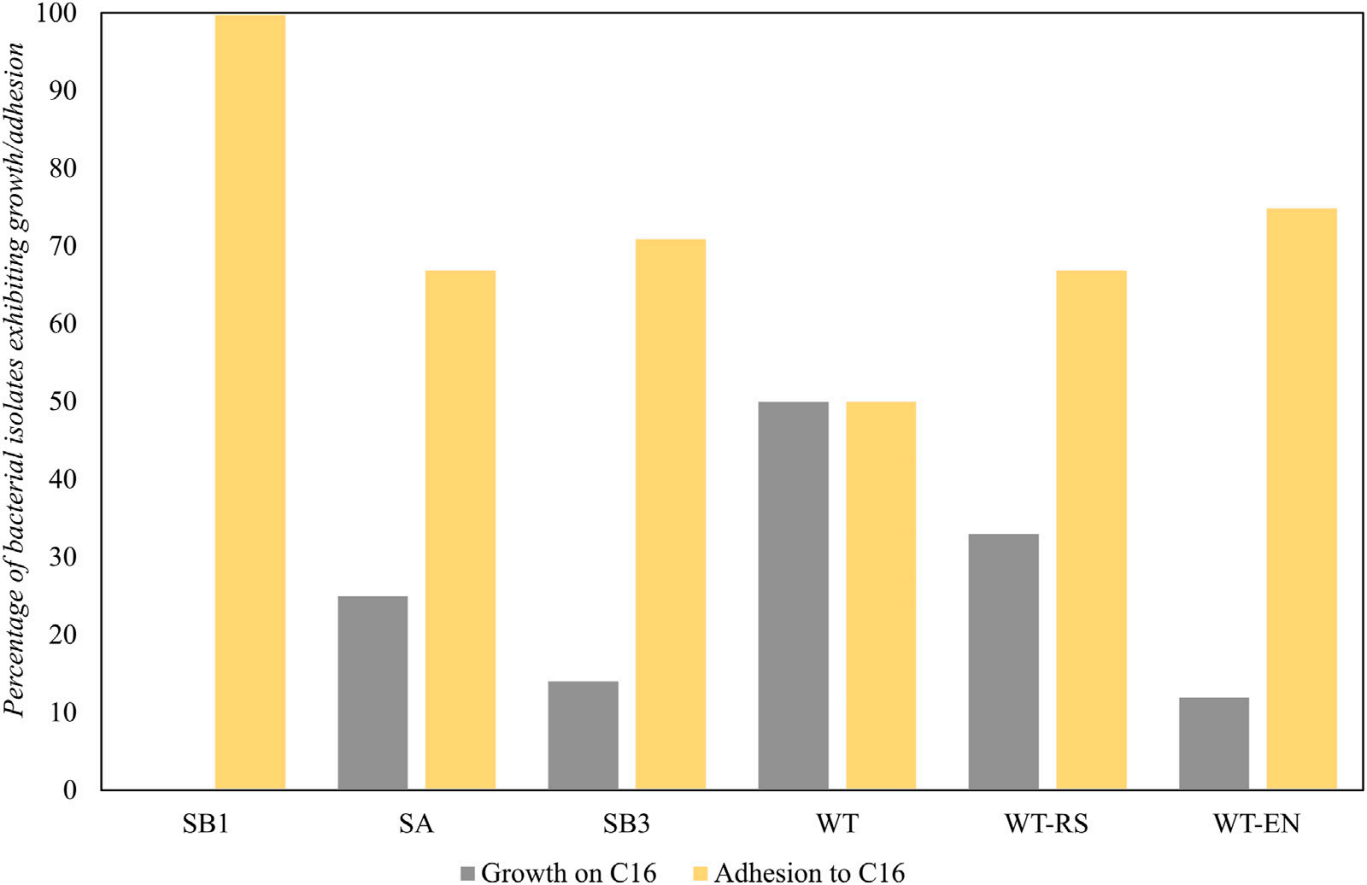
Bacterial growth and adhesion to hexadecane of strains isolated from samples SB1, SA, SB3, WT, WT-RS and WT-EN. The results of the BATH test were expressed as capacity to adhere to a hydrophobic substrate.

The ability to use hexadecane as the sole carbon and energy source was expressed in 15 strains isolated from WT soil (50%) and WT-RS rhizosphere soil (33%) associated to *S. sylvaticus* (Figure 8). This indicated that the plant environment was favorable for the recruitment of hydrocarbon-degrading strains.

All the strains isolated from the oil spill point SB1 showed hexadecane adhesion, due to a possible selective pressure in the presence of high hydrocarbon concentrations. In the rhizosphere of *S. sylvaticus*, the number of isolated strains able to adhere to hexadecane increased from WT to WT-RS and WT-EN fractions. Adhesion processes are fundamental for bacterial colonization of plants as well as for biodegradation of TPH. According to our findings, proximity to *S. sylvaticus* roots was a positive factor for recruiting bacteria with adhesion ability to hexadecane and functional genes for hydrocarbon degradation. These data are in accordance with Fatima *et al.* (Fatima et al., 2018), who proved that plant endophytes were more efficient in colonizing root endosphere than ectophere and in expressing functional genes involved in petroleum hydrocarbon degradation. In line with this, our qPCR results confirmed that a higher number of functional biomarkers were present in the plant proximity. Furthermore, 27% of endophytic bacteria were able to grow with 32 mg L^-1^ of Zn, thus evidencing resistance to this heavy metal.

The ability to adhere to hexadecane may be related to the production of biosurfactants, useful to increase the bioavailability of the contaminants, ensuring a more successful biodegradation. This might also play a role in root adhesion. Zhou et al. (2020) demonstrated that the biosurfactants produced by bacteria reduce the surface tension, promoting high pollutant-displacement efficiency. This results in the successful degradation of petroleum hydrocarbons. Aside from biodegradation capabilities, metal tolerance is important in environmental isolates. In particular, the endophytes isolated form *S. sylvaticus* were resistant to Zn, found to be an inorganic contaminant at the site. This is in accordance with previous studies showing that *S. sylvaticus*, a monocotyledon belonging to the family Cyperaceae, is able to withstand periods of submersion and to grow in the presence of high concentrations of hydrocarbons and heavy metals and particularly to remove Zn (Schück and Greger, 2020).

In order to focus on bacterial traits useful to improve plant fitness in contaminated environment, the isolated strains were analyzed for their PGP characteristic (Table 5). Among the tested PGP characteristics, N_2_ fixation was a relevant ability of bacterial strains isolated from rhizosphere soils, evidencing the recruitment of beneficial bacteria by the plant. The ability to solubilize phosphate was present in bacterial strains isolated from rhizo-endosphere only, evidencing that the plant has exerted a high selection of this trait inside the roots in accordance with literature (Sarmah and Sarma, 2023). IAA production was the most common PGP trait in the isolates. IAA is a phytohormone that plays a central role in root elongation, especially root hairs and secondary roots, which consequently leads to an increase in root exudates, facilitating the absorption of minerals from the soil (Bennis et al., 2022) and recruitment of beneficial bacteria (Bennis et al., 2022). Almost all the endophytic strains were able to produce siderophores. In anoxic environments as the wetland where *S. sylvaticus* was sampled, iron may not be limiting, but the aerenchima of the roots may cause its oxidation and precipitation (Liu et al., 2017). In such iron-limiting conditions, the plant selects for bacteria that produce siderophores in order to increase its bioavailability. EPS producing strains were mostly present at the wetland paddy soil, where they might exert the functional role of improving soil aggregation (Costa et al., 2018).

**TABLE 5:** PGP traits of bacterial strains isolated from samples SA, WT, WT-RS and WT-EN. Data are expressed as percentage of strains that showed positive response for each trait in each sample.

	SA	WT	WT-RS	WT-EN
N_2_ fixation	83	0	67	33
Inorganic P Solubilization	0	0	0	52
IAA production	42	67	67	60
Siderophore production	42	0	67	95
EPS Production	8	50	0	32
Motility	25	67	33	100

All endophytic strains were positive to the motility test, while strains isolated from other compartments were less motile. Motility as well as the ability to adhere to the root surface is an essential phenotype in root colonization (Santaella et al., 2008). The WT-EN strains were the best performing in terms of presence of PGP traits, compared to the strains isolated from the other samples.

The presence of PGP characteristics in the strains isolated from *S. sylvaticus* indicates that the plant could recruit bacteria able to promote its growth.

### *In vivo* germination promotion

**FIGURE 9:**
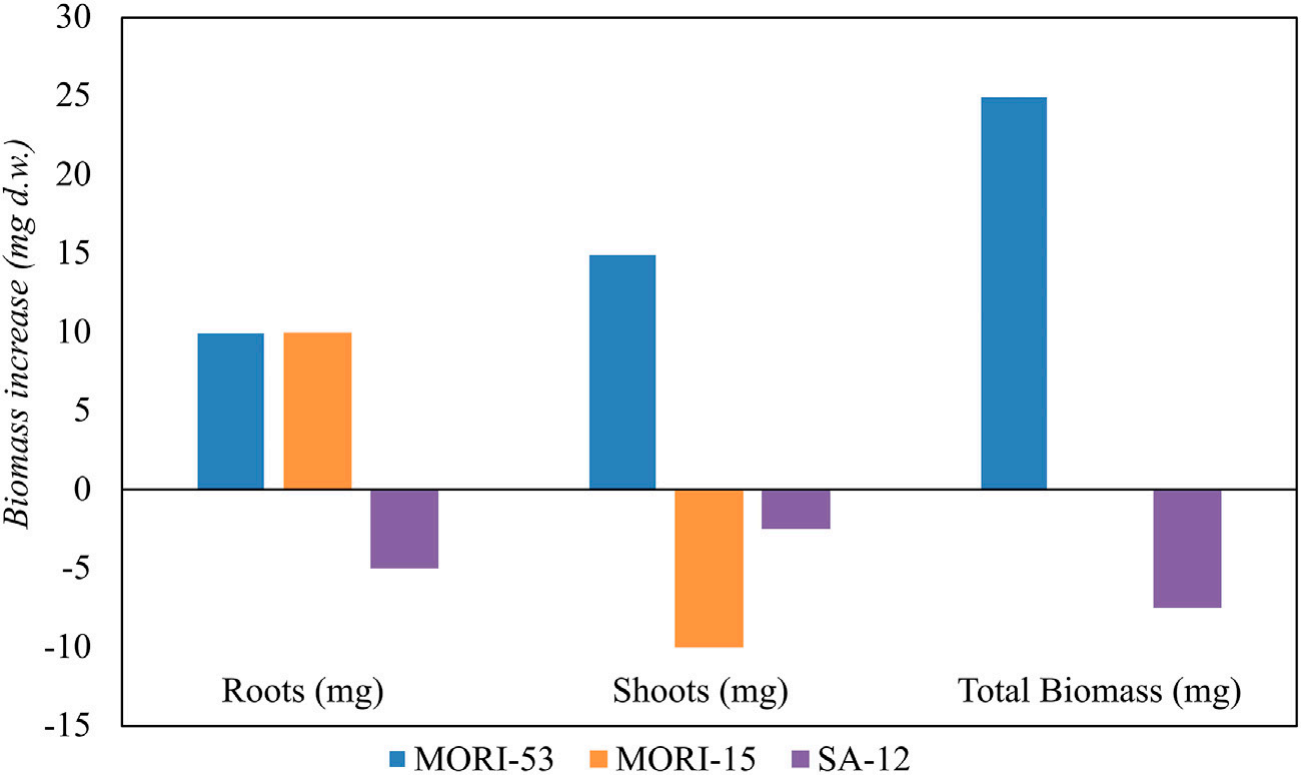
Effect of inoculation of PGPR strains (*Aeromonas media* strain MORI-53, *Pseudomonas koreensis* MORI-15 and *Rhodococcus kronopolitis* SA-12) on *Zea mays* biomass production (mg). Biomass was calculated by subtracting dry weights of roots, shoots and total biomass of bacterized plants with the respective uninoculated controls.

Based on hydrocarbon degradation ability, Zn resistance and PGP characteristics, the endophytic strains *A. media* strain MORI-53, *P. koreensis* strain MORI-15, and *R. kronopolitis* strain SA-12 were chosen within the 93 bacterial strains collection to test plant growth promotion in growth pouches experiments. After 28 days of incubation (Figure 9), *A. media* strain MORI-53 induced a higher root and shoot biomass in *Z. mays* compared to the other tested stains and to the uninoculated control (Supplementary Table S8). *Pseudomonas koreensis* strain MORI-15 and *R. kronopolitis* strain SA-12 did not promote plant growth with respect to the control.

*Aeromonas media* strain MORI-53 was also able to promote *O. sativa* growth, with a 50% increase in the total biomass dry weight compared to the uninoculated control. The significant increase of root biomass also in rice confirmed the strain PGP abilities.

Different studies confirm the efficiency of endosphere and rhizosphere bioinoculation on seeds germination (Iutynska et al., 2022), with positive effects on root development and increased dry weight (Chatterjee et al., 2017).

## Conclusion

Oil-spill affected the protected area, as demonstrated by the shift in diatom population structure and morphology. In order to preserve ecosystem functioning in the site (*i.e.*, groundwater and ditches protection within an SCI, agricultural and recreative activities), a sustainable approach as bacterial-assisted phytoremediation must be taken into account. The present work demonstrated that both indigenous bacteria and plants can withstand contamination and participate to a natural attenuation process. Beside this, the use of either crop or local plant species in association with the tested hydrocarbon-degrading PGPR could determine a considerable removal of hydrocarbons, also considering the large volume of soil likely explored by plant roots.

Future mesocosm experiments conducted on both *Z. mays* and *Scirpus* species will define how the interaction between PGP bioinoculants (used as individual strains or consortia) and plants could determine a significant improvement of rhizodegradation in a close-to-real scenario. The use of both crop and native plants might avoid issues such as adaptability to soil or climate conditions. Moreover, crop species might have the advantage of being managed easily by farmers in case of a large scale phytoremediation intervention. Above all, a rigorous monitoring plan must be developed to prevent any loss of intervention efficiency and to address potential failures.

## Data Availability

The datasets presented in this study can be found in online repositories. The names of the repository and accession numbers can be found at UNIMI Dataverse: https://dataverse.unimi.it/dataverse/HMBV.
