# Supplementary material for: Ecological indicators and biological resources for hydrocarbon rhizoremediation in a protected area
Source: Front Bioeng Biotechnol. 2024 Apr 12;12:1379947. doi: 10.3389/fbioe.2024.1379947 (PMC11046468; doi:10.3389/fbioe.2024.1379947)

**Ecological indicators and biological resources for hydrocarbon rhizoremediation in a protected area/site**

**Alice Melzi^1^, Sarah Zecchin^1^, Stefano Gomarasca^2^, Alessandro Abruzzese^3^ and Lucia Cavalca^1^***

*^1^Dipartimento di Scienze per gli Alimenti, la Nutrizione e l'Ambiente (DeFENS), Università degli Studi di Milano, Via Celoria 2, I-20133, Milano, Italy*

*^2^Dipartimento di Scienze e Politiche Ambientali (ESP), Università degli Studi di Milano, Via Celoria 2, I-20133 Milano, Italy;*

*^3^Dipartimento di Scienze Agrarie e Ambientali (DISAA), Università degli Studi di Milano, Via Celoria 2, I-20133 Milano, Italy;*

***Correspondence:**

Corresponding Author

[lucia.cavalca@unimi.it](mailto:lucia.cavalca@unimi.it)

**Keywords**: **PGPR, Rhizodegradation, Diatoms, *Ulnaria*, Protected Areas Restoration, Bioremediation, Total Petroleum Hydrocarbons**

**Supplementary materials**

TABLE S1.Samples and analyses carried out in the present work

| Samples name | Sample type | Analysis performed |
| --- | --- | --- |
| ST1 | Sediment | - Chemical characterization - Diatom population |
| ST2 |  |  |
| ST3 |  |  |
| ST4 |  |  |
| SB1  SB2 | Subsoil | - Chemical characterization - Isolation of bacteria - Hydrocarbon biodegradation in soil slurry - Functional genes in environmental DNA |
| SA | Vegetated soil | - Chemical characterization - Isolation of bacteria - Hydrocarbon biodegradation in soil slurry - Functional genes in environmental DNA |
| SB3 | Vegetated soil | - Chemical characterization - Isolation of bacteria - Hydrocarbon biodegradation in soil slurry - Functional genes in environmental DNA - Phytotoxicity test in mesocosm |
| WT | Vegetated soil | - Chemical characterization - Isolation of bacteria - Functional genes in environmental DNA - Phytotoxicity test in mesocosm |
| PZ1 | Groundwater | - Chemical characterization |
| PZ2 |  |  |
| PZ3 |  |  |

TABLE S2. Macro- and micro-elements total concentrations detected through ICP-MS and ICP-OES analysis performed on the sampled soils and sediments, and their law limits (imposed by Dlgs 152/2006 and Directive 2000/60/EC). For sediments Na, Mg, K and Ca were not evaluated. Se and Mo were below the sensitivity threshold of the analysis tool. ul = undefined legal limits.

| **Element**  **mg kg^-1^ dw** | **Sediments samples** | | | | | | | |  | **Soil samples** | | | | | | | | | | **Law limits**  **Directive 2000/60/EC** |
| --- | --- | --- | --- | --- | --- | --- | --- | --- | --- | --- | --- | --- | --- | --- | --- | --- | --- | --- | --- | --- |
|  | **ST1** | RTD% | **ST2** | RTD% | **ST3** | RTD% | **ST4** | RTD% |  | **SB1** | RTD% | **SB2** | RTD% | **SA** | RTD% | **SB3** | RTD% | **WT** | RTD% |  |
| **Mn** | 188 | 0.12 | 2948 | 0.49 | 3312 | 0.58 | 347 | 0.35 |  | 228 | 0.13 | 258 | 0.2 | 478 | 0.28 | 258 | 0.11 | 211 | 0.21 | ul |
| **Fe** | 10316 | 0.97 | 42157 | 0.27 | 49762 | 0.11 | 14740 | 0.51 |  | 13132 | 0.9 | 14170 | 0.89 | 10589 | 0.87 | 12521 | 0.56 | 18826 | 0.9 | ul |
| **Co** | 9.7 | 0.99 | 8.6 | 0.31 | 8.9 | 0.24 | 8 | 0.52 |  | 9.2 | 0.1 | 7.3 | 0.12 | 3.4 | 0.87 | 4.5 | 0.55 | 6.7 | 0.21 | 20 |
| **Ni** | 43.4 | 0.25 | 40 | 0.38 | 37 | 0.51 | 39 | 0.39 |  | 0.03 | 0.22 | 0.03 | 0.24 | 0.03 | 0.13 | 0.03 | 0.11 | 0.03 | 0.27 | 120 |
| **Cu** | 18.5 | 1.01 | 16.6 | 0.63 | 14 | 0.53 | 15.6 | 0.83 |  | 2.6 | 0.99 | 1.2 | 0.98 | 16.6 | 0.89 | 1.57 | 0.97 | 18.8 | 0.1 | 120 |
| **Zn** | 97.3 | 0.37 | 91.8 | 0.47 | 88 | 0.55 | 92 | 0.29 |  | 34.4 | 0.23 | 32.2 | 0.21 | 45.7 | 0.22 | 32.3 | 0.34 | **158** | 0.23 | 150 |
| **As** | 1.48 | 22.2 | **72** | 0.74 | **69** | 0.69 | 0.98 | 1.22 |  | 3.37 | 0.12 | 5.02 | 0.12 | 7 | 0.24 | 5.1 | 0.27 | 14.8 | 0.9 | 20 |
| **Se** | - |  | - |  | - |  | - |  |  | 0.38 | 0.9 | 0.64 | 0.89 | 0.22 | 0.87 | 0.75 | 0.12 | 0.25 | 0.23 | 3 |
| **Mo** | - |  | - |  | - |  | - |  |  | 0.7 | 0.23 | 1.05 | 0.56 | 1.09 | 0.43 | 1.5 | 0.67 | 1.4 | 0.87 | ul |
| **Cd** | 0.7 | 1.58 | 0.9 | 0.82 | 1.3 | 0.72 | 0.6 | 0.92 |  | 1.03 | 0.28 | 1.05 | 0.11 | 0.82 | 0.9 | 1.2 | 0.45 | 1 | 0.32 | 2 |
| **Pb** | 6.7 | 1.22 | 11 | 1.33 | 12 | 1.41 | 8 | 0.83 |  | 3.92 | 0.34 | 4.6 | 0.35 | 41.3 | 0.36 | 10.6 | 0.34 | 66.6 | 0.35 | 120 |
| **Al** | 12066 | 0.48 | 10658 | 1.43 | 11892 | 1.68 | 10076 | 1.22 |  | 11751 | 0.22 | 11887 | 0.24 | 11945 | 0.45 | 13749 | 0.34 | 29436 | 0.35 | ul |
| **Cr** | 41 | 0.37 | 39 | 0.39 | 37 | 0.46 | 36 | 0.28 |  | 35.9 | 0.23 | 25 | 0.25 | 21.5 | 0.27 | 20.6 | 0.11 | 47.6 | 0.23 | 150 |
| **Na** | - |  | - |  | - |  | - |  |  | 342 | 0.12 | 325.2 | 0.13 | 172 | 0.15 | 159 | 0.12 | 257 | 0.1 | ul |
| **Mg** | - |  | - |  | - |  | - |  |  | 4993 | 0.9 | 4181 | 0.89 | 2772 | 1.01 | 3609 | 0.97 | 5601 | 0.9 | ul |
| **K** | - |  | - |  | - |  | - |  |  | 1998 | 0.9 | 2447 | 0.98 | 1461 | 0.99 | 1752 | 1.02 | 4242 | 1.22 | ul |
| **Ca** | - |  | - |  | - |  | - |  |  | 3547 | 1.2 | 2732 | 1.2 | 11111 | 1.03 | 2582 | 1.23 | 7025 | 1.2 | ul |
| **P** | 753 | 0.69 | 1683 | 0.59 | 1702 | 0.63 | 1261 | 0.55 |  | 265 | 0.99 | 292 | 0.98 | 586 | 0.98 | 268 | 0.87 | 705 | 0.78 | ul |

TABLE S3. Physico-chemical characteristics of groundwater samples.

| Samples | Sampling period | Fe (II) | Fe(III) | Mn (II) | Mn (III) | Toluene | MtBE | HC TOT | pH | Conductivity | Pot. Red/OX |
| --- | --- | --- | --- | --- | --- | --- | --- | --- | --- | --- | --- |
|  |  | µg/L | µg/L | µg/L | µg/L | µg/L | µg/L | µg/L |  | µS/cm | mV |
| PZ1 | 05/17 | 2.5±1.2 | 48±7.27 | 382±9.1 | 30±3.2 | <0.1±0.018 | 3.3±1 | <15±2.21 | 7±0.5 | 270±13.5 | 118±1.1 |
|  | 08/17 | < 2±1.1 | 14±3.1 | 524±14 | 30±3.2 | <0.1±0.018 | 4.6±2.1 | <15±2.3 | 6.7±0.3 | 295±14.5 | -64±2.2 |
|  | 10/17 | < 2±1.4 | 12±2.56 | 541±15 | 22±2.7 | <0.1±0.023 | <0.1±0.024 | <15±3.1 | 6.7±0.1 | 270±27 | 57±3.2 |
|  | 03/18 | < 2±1 | 28±5.5 | 740±23 | 65±9.71 | <0.1±0.018 | 0.6±0.43 | <15±2.7 | 6.8±0.2 | 290±14.5 | 66±4.3 |
|  | 05/18 | < 2±1 | 80±9.2 | 601±21 | 12±1.1 | <0.1±0.018 | 2±2.2 | <15±4 | 6.5±0.3 | 518±17 | 5.5±1.56 |
|  | 07/18 | < 2±1 | 8±1.2 | 405±17.4 | 12±3.2 | <0.1±0.017 | <0.1±0.019 | <15±2.6 | 7.8±0.4 | 374±23.1 | -109±5 |
|  | 09/18 | < 2±1.8 | 28±5.7 | 526±21 | 48±5.12 | <0.1±0.012 | <0.1±0.009 | <15±2.7 | 7.7±0.2 | 140±14.5 | 118±2.2 |
|  | 03/19 | < 2±1 | 32±6.23 | 426±16 | 42±4.45 | 0.32±0.065 | <0.1±0.021 | <15±3.1 | 7.2±0.3 | 315±24.2 | 57±3.5 |
|  | 05/19 | < 2±1.5 | 6.1±1.1 | 382±13 | 21±4.5 | <0.1±0.014 | 0.11±0.022 | <15±3.5 | 6.6±0.2 | 350±25 | -67±2.3 |
| PZ 2 | 05/17 | 19±11 | 111±6.3 | 43±6.7 | 8±1.2 | <0.1±0.018 | <0.1±0.011 | 20793±45 | 6.4±0.1 | 280±17.2 | 40±3.5 |
|  | 08/17 | < 2±1 | 116±7.1 | 66±4.5 | 8±1.2 | <0.1±0.016 | 34.1±4.2 | <15±2.3 | 6.7±0.3 | 295±12 | 237±3.2 |
|  | 10/17 | < 2±1.1 | 22±3 | <2±2.2 | 1.8±0.3 | <0.1±0.018 | <0.1±0.01 | <15±2.1 | 7.3±0.1 | 270±13.2 | 180±4.5 |
|  | 03/18 | 25±21 | 417±7.8 | 358±8.1 | 59±5.1 | <0.1±0.017 | 107±9.2 | 1297±51 | 7.5±0.4 | 200±10.2 | 70±6.7 |
|  | 05/18 | 11±9 | 293±9.1 | 479±8 | 32±3.2 | <0.1±0.018 | 2.6±0.43 | 212±11 | 7.1±0.2 | 278±15 | 170±23 |
|  | 07/18 | < 2±1 | 17.1±3.2 | <2±1.3 | 1.7±0.03 | <0.1±0.021 | <0.1±0.019 | 274±25 | 6.5±0.21 | 340±21.4 | 55±13.4 |
|  | 09/18 | < 2±1 | 182±8 | 1.7±0.9 | 9.1±1.15 | <0.1±0.018 | <0.1±0.015 | <15±2.5 | 7.6±0.24 | 180±12.2 | 85±9.87 |
|  | 03/19 | 28.1±12 | 482±8.1 | 861±11 | 15±2.4 | <0.1±0.015 | 3.8±1.1 | 16395±155 | 8±0.31 | 617±15.3 | -75±4.5 |
|  | 05/19 | 11162±31 | 7554±35 | 1571±46 | 162±9.87 | <0.1±0.018 | <0.1±0.015 | 9131±87 | 7±0.01 | 368±15.1 | -33±3.6 |
| PZ 3 | 05/17 | 26.8±23 | 679±12 | 647±12 | 159±11 | <0.1±0.016 | 67±4.5 | 98±11 | 7.3±0.3 | 355±17.3 | 40±4.5 |
|  | 08/17 | < 2±1.5 | 117±7 | 218±6.3 | 15±2.5 | <0.1±0.018 | <0.1±0.012 | <15±2.6 | 6.8±0.2 | 240±23 | 230±6.78 |
|  | 10/17 | 16.9±13 | 974±43 | 286±7 | 38±1,7 | <0.1±0.018 | <0.1±0.021 | <15±3.5 | 7.2±0.3 | 280±13 | 154±9.8 |
|  | 03/18 | < 2±1.1 | 71±14 | 2075±25 | 52±6.7 | <0.1±0.020 | 2.1±0.1 | <15±2.1 | 7±0.1 | 300±15 | 74±5.6 |
|  | 05/18 | < 2±1 | 241±31 | 771±31 | 9±1.4 | <0.1±0.018 | <0.1±0.016 | <15±2.2 | 6.8±0.17 | 300±17.8 | 180±6.,7 |
|  | 07/18 | < 2±1 | 5.9±3.2 | 93±15.6 | 4.5±0.98 | <0.1±0.018 | <0.1±0.016 | <15±2.1 | 7±0.18 | 340±18 | -100±2.34 |
|  | 09/18 | < 2±1.6 | 54±8.9 | 147±12 | 9±1.9 | <0.1±0.018 | <0.1±0.019 | <15±2.1 | 7.8±0.2 | 193±13.4 | 80±5.6 |
|  | 03/19 | < 2±1.7 | 24±5.3 | 13±0.11 | 2.1±0.11 | <0.1±0.017 | <0.1±0.020 | <15±2.6 | 7±0.01 | 312±16.7 | 70.2±5.5 |
|  | 05/19 | < 2±1.3 | 59±11 | 92±12 | 9.2±1.3 | <0.1±0.019 | <0.1±0.021 | <15±3.2 | 7±0.01 | 370±18.3 | 12±1.2 |
| ST1 | 05/17 | ND | ND | ND | ND | <0.1±0.01 | <0.1±0.021 | <0.15±2.9 | ND | ND | ND |
|  | 08/17 | ND | ND | ND | ND | <0.1±0.018 | <0.1±0.019 | <0.15±2.8 | ND | ND | ND |
|  | 10/17 | ND | ND | ND | ND | <0.1±0.017 | <0.1±0.019 | <0.15±3.2 | ND | ND | ND |
|  | 03/18 | ND | ND | ND | ND | <0.1±0.019 | <0.1±0.016 | <0.15±3 | ND | ND | ND |
|  | 05/18 | ND | ND | ND | ND | <0.1±0.019 | <0.1±0.016 | <0.15±2.9 | ND | ND | ND |
|  | 07/18 | ND | ND | ND | ND | <0.1±0.019 | <0.1±0.016 | <0.15±2.8 | ND | ND | ND |
|  | 09/18 | ND | ND | ND | ND | <0.1±0.021 | <0.1±0.016 | <0.15±3 | ND | ND | ND |
|  | 03/19 | ND | ND | ND | ND | <0.1±0.017 | <0.1±0.017 | <0.15±2.5 | ND | ND | ND |
|  | 05/19 | ND | ND | ND | ND | <0.1±0.019 | <0.1±0.01 | <0.15±2.5 | ND | ND | ND |
| ST2 | 05/17 | ND | ND | ND | ND | 2.2±0.1 | 1±0.021 | <0.15±3 | ND | ND | ND |
|  | 08/17 | ND | ND | ND | ND | <0.1±0.017 | <0.1±0.021 | <0.15±3.1 | ND | ND | ND |
|  | 10/17 | ND | ND | ND | ND | <0.1±0.019 | <0.1±0.021 | <0.15±3.1 | ND | ND | ND |
|  | 03/18 | ND | ND | ND | ND | <0.1±0.019 | 0.6±0.016 | 77±32 | ND | ND | ND |
|  | 05/18 | ND | ND | ND | ND | <0.1±0.018 | 0.7±0.016 | <0.15±3.1 | ND | ND | ND |
|  | 07/18 | ND | ND | ND | ND | <0.1±0.018 | <0.1±0.013 | <0.15±3.2 | ND | ND | ND |
|  | 09/18 | ND | ND | ND | ND | <0.1±0.016 | <0.1±0.019 | <0.15±3.2 | ND | ND | ND |
|  | 03/19 | ND | ND | ND | ND | <0.1±0.018 | <0.1±0.019 | <0.15±2.8 | ND | ND | ND |
|  | 05/19 | ND | ND | ND | ND | <0.1±0.019 | 0.2±0.013 | <0.15±3.2 | ND | ND | ND |
| ST3 | 05/17 | ND | ND | ND | ND | <0.1±0.019 | 35±0.45 | <0.15±2.9 | ND | ND | ND |
|  | 08/17 | ND | ND | ND | ND | <0.1±0.019 | 0.3±0.019 | <0.15±3.2 | ND | ND | ND |
|  | 10/17 | ND | ND | ND | ND | <0.1±0.021 | <0.1±0.019 | <0.15±3.2 | ND | ND | ND |
|  | 03/18 | ND | ND | ND | ND | <0.1±0.02 | 1.3±0.2 | <0.15±2.9 | ND | ND | ND |
|  | 05/18 | ND | ND | ND | ND | <0.1±0.022 | <0.1±0.019 | <0.15±3.2 | ND | ND | ND |
|  | 07/18 | ND | ND | ND | ND | <0.1±0.022 | <0.1±0.017 | <0.15±3.2 | ND | ND | ND |
|  | 09/18 | ND | ND | ND | ND | <0.1±0.019 | <0.1±0.016 | <0.15±3 | ND | ND | ND |
|  | 03/19 | ND | ND | ND | ND | <0.1±0.019 | <0.1±0.016 | <0.15±3.1 | ND | ND | ND |
|  | 05/19 | ND | ND | ND | ND | <0.1±0.017 | <0.1±0.015 | <0.15±3.2 | ND | ND | ND |
| ST4 | 05/17 | ND | ND | ND | ND | <0.1±0.017 | <0.1±0.019 | <0.15±2.9 | ND | ND | ND |
|  | 08/17 | ND | ND | ND | ND | <0.1±0.016 | 0.5±0.019 | <0.15±3 | ND | ND | ND |
|  | 10/17 | ND | ND | ND | ND | <0.1±0.014 | <0.1±0.02 | <0.15±3.1 | ND | ND | ND |
|  | 03/18 | ND | ND | ND | ND | <0.1±0.015 | <0.1±0.019 | <0.15±3.2 | ND | ND | ND |
|  | 05/18 | ND | ND | ND | ND | <0.1±0.016 | <0.1±0.019 | <0.15±3.2 | ND | ND | ND |
|  | 07/18 | ND | ND | ND | ND | <0.1±0.019 | <0.1±0.018 | <0.15±2.7 | ND | ND | ND |
|  | 09/18 | ND | ND | ND | ND | <0.1±0.021 | <0.1±0.018 | <0.15±3.5 | ND | ND | ND |
|  | 03/19 | ND | ND | ND | ND | <0.1±0.02 | <0.1±0.018 | <0.15±3 | ND | ND | ND |
|  | 05/19 | ND | ND | ND | ND | <0.1±0.019 | <0.1±0.019 | <0.15±3.2 | ND | ND | ND |

*ND., not determined.*

TABLE S4. Physico-chemical characteristics of the sampled soils.

| Samples | SB1 | SB2 | SA | SB3 | WT |
| --- | --- | --- | --- | --- | --- |
| Depth of sampling  (u.m. (m)) | 4 m  b.g. | 4.5 m  b.g. | 5 cm | 1.5 m  b.g. | 1 m  b.g. |
| Texture | sandy  loam | loamy  sandy  clay | sandy  loam | sandy  loam | loamy  sandy  clay |
| pH | 7±0.5 | 7.1±0.3 | 6.5±0.45 | 6±0.45 | 6±0.2 |
| TOC  (g kg^-1^ d.w) | 5.6±0.33 | 1.6±0.31 | 210±0.21 | 11±0.12 | 84±0.11 |
| CEC  (cmol_(+)_ kg^-1^ d.w.) | 3.8±0.45 | 5.6±0.48 | 32±0.11 | 12±0.12 | 53.4±0.13 |
| TKN  (mg kg^-1^ d.w.) | 0.25±0.13 | 0.16±0.11 | 4±0.12 | 0.65±0.9 | 4.54±0.9 |
| C/N | 22.5±0.12 | 9.9±0.12 | 19±0.11 | 16.3±0.13 | 18.5±0.12 |
| P_2_O_5_  (g kg^-1^ d.w.) | 41.3±0.35 | 41±0.34 | 12±0.37 | 5.81±0.45 | 18.3±0.47 |

b.g. below ground

CEC, cation exchange capacity

TKN, Total Kjeldahl Nitrogen

TABLE S5a. Total species of diatom found in the ST1, ST2, ST3 and ST4.

|  | **ST1** | **ST1** | **ST1** | **ST1** | **ST1** | **ST2** | **ST2** | **ST2** | **ST2** | **ST2** | **ST3** | **ST3** | **ST3** | **ST3** | **ST3** | **ST4** | **ST4** | **ST4** | **ST4** | **ST4** |  | |
| --- | --- | --- | --- | --- | --- | --- | --- | --- | --- | --- | --- | --- | --- | --- | --- | --- | --- | --- | --- | --- | --- | --- |
|  | Aug-2017 | May-2018 | Aug-2018 | June-2019 | Sept-2019 | Aug-2017 | May-2018 | Aug-2018 | June-2019 | Sept-2019 | Aug-2017 | May-2018 | Aug-2018 | June-2019 | Sept-2019 | Aug-2017 | May-2018 | Aug-2018 | June-2019 | Sept-2019 |  |  |
| **species** | frustules | frustules | frustules | frustules | frustules | frustules | frustules | frustules | frustules | frustules | frustules | frustules | frustules | frustules | frustules | frustules | frustules | frustules | frustules | frustules | **Total** | |
| *Achnanthes exigua* var. *elliptica*Hustedt | - | - | - | - | - | - | - | - | - | - | - | - | - | - | - | - | - | - | 1 | - | **1** | |
| *Achnanthes lutherii* Hustedt | - | - | - | - | - | - | - | - | - | - | - | - | - | - | - | 7 | - | - | - | - | **7** | |
| *Achnanthes minutissima* Kutzing var. *jackii* (Rabenhorst) Lange-Bertalot | - | - | - | 7 | 9 | - | - | - | - | - | - | - | - | 15 | 3 | - | - | - | - | - | **34** | |
| *Achnanthes petersenii* Hustedt | - | - | 4 | - | - | - | - | - | - | - | - | - | - | - | - | - | - | - | - | - | **4** | |
| *Achnanthes rupestoides* Hohn | 8 | - | 7 | 2 | 2 | 3 | 2 | 3 | 2 | - | 7 | - | - | - | - | 3 | - | 5 | - | - | **44** | |
| *Achnanthidium biasolettianum* (Grunow) Lange-Bertalot | - | - | 18 | - | - | - | - | - | - | - | 1 | 8 | 7 | - | - | 4 | 129 | 21 | - | 5 | **193** | |
| *Achnanthidium* *affine* (Grun) Czarnecki | - | - | - | - | - | - | - | - | - | - | - | - | - | - | - | - | - | - | - | 5 | **5** | |
| *Achnanthidium alteragracillima* (Lange-Bertalot) Round Bukhtiyarova | - | - | - | - | 9 | - | - | - | - | - | - | - | - | - | - | - | - | - | - | - | **9** | |
| *Achnanthidium lineare* W.Smith | 162 | 276 | 166 | 253 | 168 | 8 | 5 | - | 25 | - | 52 | 22 | 49 | 13 | 44 | 125 | 93 | 165 | 68 | 138 | **1832** | |
| *Achnanthidium minutissimum* (Kützing) Czarnecki | 20 | 6 | 13 | 8 | 49 | - | - | - | - | - | 11 | 29 | 11 | 7 | 4 | 8 | 18 | - | 89 | 44 | **317** | |
| *Achnanthidium straubianum* (Langhe Bertalot) Langhe Bertalot | - | - | - | - | - | 3 | - | - | 5 | 3 | - | - | - | - | - | - | - | - | - | - | **11** | |
| *Amphipleura pellucida* Kützing | 5 | 8 | 2 | 1 | 6 | - | - | - | - | - | - | - | - | - | - | 8 | 2 | 16 | 5 | 13 | **66** | |
| *Amphora holsatica* Hustedt | - | - | - | 2 | 2 | - | - | - | 3 | - | - | - | - | - | - | - | - | - | - | - | **7** | |
| *Amphora inariensis* Krammer | 1 | 12 | 6 | 1 | - | - | 13 | - | - | - | - | 7 | - | - | - | 4 | 7 | 5 | - | 2 | **58** | |
| *Amphora normanii Rabenhorst* | 1 | - | - | - | - | - | - | - | - | - | - | - | - | - | - | - | - | - | - | - | **1** | |
| *Amphora ovalis* (Kützing) Kützing | 1 | 3 | 2 | 5 | 6 | 5 | 3 | 2 | 2 | 1 | - | 2 | 1 | - | - | 4 | 2 | - | 3 | 1 | **43** | |
| *Amphora pediculus* (Kützing) Grunow | 3 | - | - | 2 | - | 1 | - | - | 8 | - | 3 | - | - | 8 | 1 | 3 | 2 | - | - | 2 | **33** | |
| *Aneumastus stroesei (*Ostrup) Mann | - | - | - | 1 | - | - | - | - | - | - | - | - | - | - | - | - | - | - | - | - | **1** | |
| *Brachysira vitrea* (Grunow) Ross | 3 | - | - | - | - | - | - | - | - | - | - | - | - | - | - | - | - | - | - | - | **3** | |
| *Caloneis lancettula(Schulz)* Langhe Bertalot e Witkowski | - | - | - | - | - | - | - | - | - | - | - | - | 2 | - | - | 1 | - | - | 1 | - | **4** | |
| *Caloneis tenuis* (Gregory) Krammer | - | - | - | - | - | - | - | - | - | - | 6 | - | - | 1 | - | - | - | - | - | - | **7** | |
| *Cocconeis euglypta* Ehrenberg | - | - | 6 | - | - | - | - | 6 | - | - | - | - | - | 12 | - | - | - | 1 | - | 3 | **28** | |
| *Cocconeis lineata* Ehrenberg | 11 | 8 | 11 | 36 | 21 | 3 | 2 | 11 | 7 | 24 | - | 8 | 14 | 23 | 11 | 28 | 4 | 28 | 56 | 15 | **321** | |
| *Cocconeis neodiminuta Krammer* | - | - | - | - | - | - | - | - | - | - | - | - | - | - | - | 4 | - | - | - | - | **4** | |
| *Cocconeis pediculus* Ehrenberg | - | - | 4 | - | - | - | - | - | - | - | - | - | 16 | - | 6 | - | - | - | - | - | **26** | |
| *Cocconeis pseudolineata* Langhe Bertalot | 7 | - | - | 1 | - | 1 | - | - | 3 | - | - | 2 | - | - | - | 8 | 3 | - | 7 | 2 | **34** | |
| *Craticula accomoda* (Hustedt) Mann | - | - | - | - | - | - | - | - | - | - | - | - | - | - | - | - | - | - | 2 | 1 | **3** | |
| *Craticula halophila (Grunow ex Van Heurck) Mann* | - | - | - | - | - | - | - | - | - | - | 3 | - | - | - | - | - | - | - | - | - | **3** | |
| *Cyclotella ocellata* Pantocsek | - | - | - | 1 | - | - | - | - | - | - | - | - | - | 5 | - | - | - | 1 | - | - | **7** | |
| *Cymbella affinis* Kützing | - | - | 6 | - | - | - | - | - | - | - | - | - | - | - | - | - | - | - | - | - | **6** | |
| *Cymbella lange-bertalotii* Krammer | - | - | - | - | - | - | - | - | - | - | - | - | - | - | - | 3 | - | - | - | - | **3** | |
| *Cymbella pusilla* Grunow in A. Schmidt al | - | - | - | 1 | - | - | - | - | - | - | - | - | - | - | - | - | - | - | - | - | **1** | |
| *Cymbella tumida* (Brébisson) Van Heurck | - | - | - | - | 1 | - | - | - | - | - | - | - | - | - | - | - | - | - | - | - | **1** | |
| *Cymbella turgidula* Grunow | - | - | - | - | - | - | 2 | - | - | - | - | - | - | - | - | - | - | - | - | - | **2** | |
| *Cymbopleura amphicephala* Krammer | - | - | - | 1 | - | - | 1 | - | - | - | - | 3 | - | 1 | - | 1 | - | - | - | 1 | **8** | |
| *Cymbopleura naviculiformis* (Auerswald) Krammer | - | - | - | - | - | - | - | - | - | - | - | - | - | - | 1 | - | - | - | - | - | **1** | |
| *Denticula tenuis* Kützing | - | - | - | - | - | - | - | - | - | - | - | - | - | - | 1 |  | - | - | - | - | **1** | |
| *Diatoma ehrenbergii* Kutzing | 1 | 2 | 6 | 1 | - | - | - | - | - | - | - | - | 1 | 2 | 3 | 1 | - | 1 | - | - | **18** | |
| *Diatoma moniliformis* Kutzing | - | - | - | - | - | - | - | 2 | - | - | 1 | - | - | 2 | - | - | - | - | - | - | **5** | |
| *Diatoma vulgaris* Bory | - | - | - | - | - | - | - | - | - | - | - | - | 1 | - | - | - | 2 | 2 | - | - | **5** | |
| *Diploneis elliptica* (Kützing) Cleve | - | - | - | - | - | - | - | - | - | - | - | - | - | - | - | - | - | - | - | 1 | **1** | |
| *Diploneis modica* Hustedt | - | - | 1 | - | - | - | - | - | - | - | - | - | - | - | - | - | - | - | - | - | **1** | |
| *Diploneis parma* Cleve | - | - | 2 | - | - | - | - | - | - | - | - | - | - | - | - | 3 | 3 | 1 | - | 3 | **12** | |
| *Diploneis peterseni* Hustedt | - | - | 1 | - | - | - | - | - | - | - | - | - | - | - | - | - | - | - | - | - | **1** | |
| *Ellerbeckia arenaria* (Moore) Crawford | - | - | - | - | 2 | - | - | - | - | - | - | - | - | - | - | - | - | - | 1 | - | **3** | |
| *Encyonema silesiacum* (Bleisch) Mann | - | - | 2 | - | 2 | - | 3 | 2 | 7 | - | - | 7 | 2 | 6 | 2 | - | - | 4 | 5 | 2 | **44** | |
| *Encyonopsis minuta* Krammer e Reichardt | - | - | 1 | - | - | - | - | - | 5 | - | - | - | - | - | 3 | - | - | - | - | 1 | **10** | |
| *Epithemia adnata* var. *minor*(Per. & Hér.-Jos.) Patrick | - | - | - | - | - | - | - | - | - | - | - | - | - | - | - | - | - | 1 | - | - | **1** | |
| *Eunotia arcus* Ehrenberg | - | - | - | - | - | - | - | 1 | - | - | - | - | - | - | - | - | - | - | - | - | **1** | |
| *Eunotia bilunaris (Ehrenberg) Schaarschmidt in Kanitz* | - | - | - | - | 1 | - | - | - | 6 | - | - | 2 | 2 | 5 | - | 1 | - | 2 | - | 1 | **20** | |
| *Eunotia minor* (Kutzing) Gronow | 1 | 5 | 2 | 1 | 3 | 14 | 11 | 133 | 34 | 43 | - | 4 | 7 | 9 | 48 | - | 2 | - | - | 7 | **324** | |
| *Eunotia monodon* Ehrenberg Drawing | - | - | - | - | - | - | - | - | - | 3 | - | - | - | - | - | - | - | - | - | - | **3** | |
| *Fallacia helensis* (Schulz) DG Mann | - | - | - | - | 5 | - | - | - | - | - | - | - | - | - | - | - | - | - | - | 2 | **7** | |
| *Fragilaria arcus* (Ehrenberg) Cleve | 5 | - | 11 | - | 3 | - | - | - | - | - | - | 7 | - | - | 2 | - | - | - | - | 1 | **29** | |
| *Fragilaria austriaca (Grunow) Lange-Bertalot* | - | - | - | - | - | - | - | - | - | - | - | - | - | - | - | 1 | - | - | - | - | **1** | |
| *Fragilaria capucina* Desmazières | 1 | - | - | - | - | - | - | - | - | - | - | - | - | 3 | - | - | - | - | - | - | **4** | |
| *Fragilaria capucina* Desmazieres var. *distans* (Grunow) Lange-Bertalot | - | - | - | - | - | - | - | - | - | - | - | - | - | 3 | - | - | - | - | 3 | 3 | **9** | |
| *Fragilaria capucina* var. *vaucheriae* (Kutzing) Lange-Bertalot | - | - | - | - | 2 | - | 2 | 1 | - | - | - | 5 | 2 | 4 | - | - | - | - | 5 | 3 | **24** | |
| *Fragilaria famelica var. famelica* (Kütz.) Lange-Bert. | - | - | - | - | - | - | - | - | - | - | - | - | - | 9 | - | - | 2 | - | - | - | **11** | |
| *Fragilaria gracilis*Østrup | - | - | - |  | - | - | - | - | - | - | - | - | - | - | - | 4 | - | - | 1 | - | **5** | |
| *Fragilaria ulna* var. *acus* (Kützing) Lange-Bertalot | 7 | - | - | 1 | 2 | - | - |  | - | - | 4 | - | - | 7 | 6 | 13 | - | - | 2 | 3 | **45** | |
| *Frustulia vulgaris* (Thwaites) De Toni | 1 | 2 | - | - | 6 | - | 2 | 1 | - | - | - | - | 1 | - | - | 3 | 2 | 4 | - | 1 | **23** | |
| *Geissleria decussis (*Oestrup) Lange-Bertalot Metzeltin | - | - | - | - | - | - | - | - | - | - | - | - | - | - | - | - | - | 1 | - | - | **1** | |
| *Gomphonema acuminatum* Ehrenberg | - | 2 | - | - | - | - | 1 | 1 | 2 | - | - | - | - | - | 3 | 3 | 2 | 6 | - | - | **20** | |
| *Gomphonema affine* Kützing | - | - | - | - | - | - | - | - | - | - | - | - | 2 | 7 | - | - | - | 3 | - | - | **12** | |
| *Gomphonema angustatum* (Kützing) Rabenhorst | - | - | - | - | - | 276 | 228 | 119 | 166 | 274 | 129 | 93 | 123 | 138 | 131 | - | - | - | 2 | 5 | **1684** | |
| *Gomphonema angustum* Agardh | - | - | - | - | - | - | - | - | - | - | - | - | - | - | - | - | - | - | 3 | - | **3** | |
| *Gomphonema clavatum* Ehrenberg | - | - | 7 | 3 | - | 19 | - | 1 | 4 | - | 7 | - | 17 | - | - | - | - | 5 | - | 4 | **67** | |
| *Gomphonema exilissimum (*Grun) Lange-Bertalot Reichardt | - | 3 | 1 | - | 1 | - | 9 | - | - | - | - | 25 | 4 | 7 | - | 3 | - | 2 | - | - | **55** | |
| *Gomphonema gracile* Ehrenberg | - | - | - | - | - | *-* | *-* | *-* | *-* | *-* | 2 | - | - | - | - | - | - | - | - | - | **2** | |
| *Gomphonema occultum*E.Reichardt & Lange-Bertalot | - | - | - | - | - | - | - | - | - | - | - | - | - | 1 | - | - | - | - | - | - | **1** | |
| *Gomphonema olivaceum* (Hornemann) Brébisson | - | - | - | - | - | - | - | - | - | - | - | - | - | - | - | - | - | - | 1 | - | **1** | |
| *Gomphonema parvulum* (Kützing) Kützing | 5 | 3 | 2 | 2 | 1 | 7 | 6 | 7 | - | - | 28 | 79 | 44 | - | - | - | 2 | 11 | - | - | **197** | |
| *Gomphonema parvulum* var. *parvulum* f. *saprophilum* Lange-Bertalot & Reichardt | - | - | - | - | - | 4 | - | - | 13 | - | 3 | - | - | 9 | - | - | - | - | - | - | **29** | |
| *Gomphonema pumilum* (Gr) Reichardt Lange-Bertalot | - | - | 7 | - | - | *-* | *-* | *-* | *-* | *-* | - | - | - | - | - | - | - | 1 | - | - | **8** | |
| *Gomphonema subclavatum* (Grunow) Grunow | - | 2 | - | - | - | - | 9 | - | - | - | - | 5 | - | - | 10 | - | 2 | - | - | - | **28** | |
| *Gyrosigma acuminatum (*Kützing) Rabenhorst | 1 | - | - | - | 3 | *-* | *-* | *-* | *-* | *-* | - | - | - | - | - | - | - | - | 2 | 1 | **7** | |
| *Hippodonta capitata* (Ehr) L-B, Metzeltin Witkowski | 1 | - | 2 | 1 | 2 | - | 1 | - | 3 | - | - | - | - | - | - | 3 | - | 3 | - | 1 | **17** | |
| *Hippodonta costulata* (Grunow)Lange-Bertalot Metzeltin Witkowski | - | - | - | - | - | *-* | *-* | *-* | *-* | *-* | - | - | - | - | - | - | - | 1 | - | 2 | **3** | |
| *Kolbesia ploenensis* (Hust) Kingston | - | - | - | - | 4 | *-* | *-* | *-* | *-* | *-* | - | - | - | - | - | - | - | - | - | - | **4** | |
| *Lemnicola exigua* (Grunow) Kulikovskiy, Witkowski & Plinski | - | - | - | - | - | - | - | - | - | - | - | - | - | - | - | - | - | - | 1 | - | **1** | |
| *Luticula goeppertiana* (Bleisch) Mann | 3 | 2 | - | - | - | - | 1 | - | - | - | - | - | - | - | - | - | - | 9 | - | - | **15** | |
| *Melosira varians* Agardh | 3 | 4 | - | 3 | - | - | - | - | 7 | - | - | - | - | 13 | 4 | 3 | 6 | - | 7 | 7 | **57** | |
| *Meridion circulare* (Greville) Agardh | - | - | - | - | - | *-* | *-* | *-* | *-* | *-* | - | - | - | - | 1 | - | - | - | 2 | - | **3** | |
| *Naidium binodis (*Ehrenberg) Husted | - | - | - | - | - | - | - | - | - | - | - | - | - | - | - | 1 | - | - | - | - | **1** | |
| *Navicula amphibola* Cleve | - | 1 | - | - | - | - | - | - | - | - | - | - | - | - | - | - | - | - | - | - | **1** | |
| *Navicula amphiceropsis* Lange-Bertalot & U.Rumrich | - | - | - | - | - | - | - | - | - | - | - | - | - | - | - | - | 1 | - | - | - | **1** | |
| *Navicula caterva* Hohn & Hellermann | - | - | - | - | - | *-* | *-* | *-* | *-* | *-* | - | 6 | - | - | - | - | - | - | - | - | **6** | |
| *Navicula constans* Hustedt | 3 | - | - | - | - | - | - | - | - | - | - | - | - | - | - | 3 | 1 | - | - | - | **7** | |
| *Navicula cryptocephala* Kützing | - | - | - | - | - | 1 | 3 | 4 | 3 | - | - | - | - | - | - | - | - | - | - | - | **11** | |
| *Navicula cryptocephala* Kützing | 27 | 5 | 11 | 10 | 15 | - | - | - | - | - | 16 | 12 | 2 | 32 | 4 | 31 | 2 | 2 | 7 | 31 | **207** | |
| *Navicula cryptotenella* Lange-Bertalot | 7 | 3 | - | - | - | - | - | - | - | - | 3 | 4 | - | - | - | 5 | 2 | - | - | - | **24** | |
| *Navicula elegans* W. Smith | - | - | - | - | - | - | - | - | - | - | - | - | - | - | - | 3 | - | - | - | - | **3** | |
| *Navicula elginensis* (W.Gregory) Ralfs | 1 | - | - | 1 | 1 | - | - | - | - | - | - | - | - | 1 | - | - | - | - | - | - | **4** | |
| *Navicula exilis* Kützing | - | - | 1 | - | - | - | - | - | - | - | - | - | - | - | - | - | - | - | - | - | **1** | |
| *Navicula gastrum (*Ehrenberg) Kützing | - | - | - | - | 1 | - | - | - | - | - | - | 1 | - | - | - | - | 2 | - | - | - | **4** | |
| *Navicula gregaria* Donkin | 1 | - | - | - | - | - | - | - | - | - | - | - | - | - | - | 1 | - | 1 | - | - | **3** | |
| *Navicula lanceolata* (Agardh) Ehrenberg | - | - | - | - | 1 | - | - | - | - | - | - | - | - | - | - | - | - | - | - | - | **1** | |
| *Navicula margalithii* Lange-Bertalot | - | - | - | - | - | - | - | - | - | - | - | - | - | - | - | 3 | - | - | - | - | **3** | |
| *Navicula menisculus* Schumann | - | - | 4 | 1 | 3 | - | 1 | - | - | - | - | - | - | - | - | - | 4 | 2 | 5 | 1 | **21** | |
| *Navicula modica*Hustedt | - | 1 | - | - | - | - | - | - | - | - | - | - | - | - | - | - | - | - | - | - | **1** | |
| *Navicula phyllepta* Kützing | - | - | - | - | - | - | - | - | - | - | - | - | - | 1 | - | - | - | - | - | - | **1** | |
| *Navicula radiosa* Kützing | 1 | - | - | 1 | - | - | - | - | - | - | - | - | - | - | - | - | - | - | 3 | - | **5** | |
| *Navicula radiosafallax* Lange-Bertalot | - | - | - | - | - | - | - | - | - | - | - | - | - | - | - | - | - | - | - | 1 | **1** | |
| *Navicula recens* (Lange-Bertalot) Lange-Bertalot | - | - | - | - | - | - | - | - | - | - | - | - | - | - | - | - | 1 | - | - | - | **1** | |
| *Navicula rostellata* Kützing | - | - | - | - | 1 | - | - | - | - | - | - | - | - | - | - | - | - | - | - | - | **1** | |
| *Navicula splendicula* Van Landingham | - | - | - | - | - | - | - | - | - | - | - | - |  | - | - | 1 | - | - | - | - | **1** | |
| *Navicula subplacentula* Hustedt | - | - | - | 1 | - | - | - | - | - | - | - | - | - | - | - | - | - | - | - | - | **1** | |
| *Navicula symmetrica R.M.Patrick* | - | - | - | - | - | - | - | - | - | - | - | - | - | - | - | - | - | - | - | 1 | **1** | |
| *Navicula tridentula* Krasske | - | - | - | - | - | - | - | - | - | - | - | - | - | - | - | - | - | 1 | - | - | **1** | |
| *Navicula tripunctata* (Müller) Bory | - | - | - | - | - | - | - | - | - | - | - | - | - | - | - | - | 1 | - | - | - | **1** | |
| *Navicula veneta* Kützing | - | - | - | - | - | 1 | - | - | 3 | - | - | - | - | - | - | - | - | - | 2 | - | **6** | |
| *Navicula viridula* (Kützing) Ehrenberg | - | - | - | - | - | - | - | - | - | - | - | - | - | 1 | - | - | - | - | - | - | **1** | |
| *Navicula viridula var. rostellata* (Kützing) Cleve | - | 2 | - | - | - | - | - | - | - | - | - | - | - | - | - | - | 1 | - | - | - | **3** | |
| *Nitzschia acicularis* (Kützing) W M Smith | 4 | - | - | - | - | - | - | - | - | - | - | - | - | - | - | 1 | - | - | - | - | **5** | |
| *Nitzschia aurariae* Cholnoky | - | - | - | - | - | - | - | - | - | - | - | - | - | - | - | - | 1 | - | - | - | **1** | |
| *Nitzschia brevissima* Grunow | - | - | - | - | - | - | - | - | - | - | - | - | - | - | - | - | 1 | - | - | - | **1** | |
| *Nitzschia dissipata* (Kützing) Grunow | 1 | - | - | - | - | - | 4 | 1 | - | - | - | - | - | - | - | 1 | 2 | - | - | - | **9** | |
| *Nitzschia diversa* Hustedt | - | - | - | - |  | - | - | - | - | - | *-* | *-* | *-* | *-* | *-* | - | - | 1 | - | - | **1** | |
| *Nitzschia fonticola* Grunow | 8 | 5 | 4 | 3 | 4 | - | 2 | 2 | - | - | 2 | - | - | - | - | - | 9 | - | 11 | 3 | **53** | |
| *Nitzschia gracilis* Hantzsch | 4 | - | - | 2 | - | - | - | - | - | - | - | - | - | - | - | - | - | - | 5 | 1 | **12** | |
| *Nitzschia linearis* (Agardh) W Smith | 1 | 4 | 2 | 1 | 3 | 3 | 2 | - | - | - | - | - | - | 2 | - | 1 | 6 | 3 | 5 | 10 | **43** | |
| *Nitzschia palea* (Kützing) W Smith | 11 | 3 | 4 | 1 | 2 | - | 1 | - | - | - | 2 | - | - | - | - | - | - | 2 | 7 | 4 | **37** | |
| *Nitzschia pellucida Grunow* | 3 | - | - | - | - | - | - | - | - | - | *-* | *-* | *-* | *-* | *-* | 7 | - | - | - | - | **10** | |
| *Nitzschia perminuta (*Grunow) M.Peragallo | 4 | 7 | - | 1 | 2 | - | - | - | - | - | 2 | 2 | - | - | - | 3 | 2 | 5 | - | 3 | **31** | |
| *Nitzschia pura* Hustedt | 3 | 6 | - | - | 4 | 1 | 1 | - | - | - | - | - | - | - | - | 3 | 3 | - | 4 | 6 | **31** | |
| *Nitzschia sublinearis* Hustedt | - | - | 2 | - | - | - | - | - | - | - | - | - | - | - | - | - | - | 2 | 1 | - | **5** | |
| *Nitzschia tubicola*Grunow | 5 | - | - | - | - | - | - | - | - | - | 6 | - | - | - | - | - | - | - | - | 1 | **12** | |
| *Pinnularia appendiculata* (C.Agardh) Schaarschmidt | - | - | 1 | - | - | - | - | - | - | - | - | - | - | - | - | - | - | - | - | - | **1** | |
| *Pinnularia gibba* Ehrenberg | 3 | - | - | - | - | - | - | - | - | - | - | - | - | - | 2 | 1 | - | - | - | - | **6** | |
| *Pinnularia interrupta* W M Smith | - | - | - | 1 | 1 | - | - | - | - | - | - | - | - | - | - | - | - | - | - | - | **2** | |
| *Pinnularia maior* (Kützing) Rabenhorst | 3 | - | - | 1 | - | - | 1 | - | 2 | - | - | 2 | - | - | - | 1 | 1 | 2 | 1 | 2 | **16** | |
| *Pinnularia saprophila* Lange-Bertalot, Kobayasi & Krammer | - | - | - | - | - | - | - | - | - | - | - | 1 | - | - | - | - | - | - | - | - | **1** | |
| *Pinnularia subcapitata W. Gregory* | - | - | - | - | - | - | - | - | - | - | 7 | - | - | - | - | - | - | - | - | - | **7** | |
| *Placoneis elginensis* (W.Gregory) E.J.Cox | - | - | 5 | - | - | - | - | - | - | - | - | - | - | - | - | - | - | 3 | - | - | **8** | |
| *Planothidium delicatulum* (Kutz) Round Bukhtiyarova | - | - | 4 | - | - | *-* | *-* | *-* | *-* | *-* | - | - | - | - | - | - | - | - | - | - | **4** | |
| *Planothidium ellipticum* (Cleve) Round Bukhtiyarova | - | - | - | 1 | - | - | - | - | - | - | - | - | - | - | - | - | - | - | - | - | **1** | |
| *Planothidium frequentissimum(Lange-Bertalot) Round & Bukht.* | 21 | 7 | 27 | 14 | 21 | 10 | - | - | 20 | 3 | 9 | - | - | 1 | - | 22 | 11 | 3 | 14 | 2 | **185** | |
| *Planothidium lanceolatum* (Kütz ex Bréb) L-B | 10 | 2 | 10 | 2 | 8 | 12 | 43 | 63 | 46 | 9 | 18 | - | - | 2 | 12 | - | - | 2 | 6 | 1 | **246** | |
| *Planothidium rostratum* (Oestrup) Lange-Bertalot | 16 | 3 | 20 | 2 | 13 | 7 | 6 | - | - | - | - | - | - | 2 | - | 51 | 15 | 41 | 32 | 29 | **237** | |
| *Platessa holsatica* (Hustedt) Lange-Bertalot | - | - | - | - | - | *-* | *-* | *-* | *-* | *-* | - | - | - | - | - | - | - | 3 | 2 | - | **5** | |
| *Psammothidium bioretii* (Germain) Bukhtiyarova et Round | - | - | - | - | - | - | - | - | - | - | - | - | - | - | 1 | - | - | - | - | - | **1** | |
| *Psammothidium oblongellum*(Oestrup) Van de Vijver | - | - | 4 | 1 | - | 3 | - | - | 5 | - | - | - | - | - | - | 1 | - | 2 | - | - | **16** | |
| *Reimeria sinuata* (Gregory) Kociolek Stoermer | 3 | - | 12 | 23 | 3 | - | - | 7 | - | - | - | - | - | - | - | - | 3 | 4 | - | 3 | **58** | |
| *Rhoicosphenia abbreviata* (Agardh) Lange-Bertalot | 5 | 5 | 2 | 2 | 6 | 1 | 2 | 2 | - | - | *-* | *-* | *-* | *-* | *-* | 3 | 42 | 24 | 17 | 7 | **118** | |
| *Sellaphora mutata* (Krasske) Lange-Bertalot | - | - | 1 | - | - | - | - | - | - | - | *-* | *-* | *-* | *-* | *-* | - | - | - | - | - | **1** | |
| *Sellaphora pupula* (Kützing) Mereschkowsky | 4 | 3 | 2 | - | 1 | - | 1 | - | 4 | - | 9 | 4 | - | - | - | 4 | 3 | 2 | 5 | 1 | **43** | |
| *Stauroneis anceps* Ehrenberg | 3 | 7 | - | - | - | 3 | 1 | - | - | - | - | - | - | - | - | - | 6 | - | - | - | **20** | |
| *Stauroneis phoenicenteron* (Nitzsch) Ehrenberg | - | - | - | - | - | - | - | - | 2 | - | - | - | - | - | - | - | - | - | - | 1 | **3** | |
| *Stauroneis prominula*(Grunow ex Cleve) Hustedt | - | - | - | - | - | - | - | - | 1 | - | - | - | - | 1 | - | - | - | - | - | - | **2** | |
| *Stauroneis smithii* Grunow | - | - | - | - | - | 1 | 1 | - | 3 | - | - | - | - | 2 | - | - | - | - | 2 | - | **9** | |
| *Stauroneis tackei* (Hustedt) Krammer Lange-Bertalot Kusber Metzeltin | - | - | - | - | - | - | - | - | - | - | - | - | - | - | - | - | - | - | - | 1 | **1** | |
| *Stauroneis wislouchii* Poretzky & Anisimova | - | - | - | - | - | - | - | - | - | - | *-* | *-* | *-* | *-* | *-* | - | - | 1 | - | - | **1** | |
| *Stauroneis kriegeri* R.M.Patrick | - | - | - | - | - | - | - | - | - | - | - | 12 | - | - | - | - | - | - | - | - | **12** | |
| *Stauroneis* *nobilis* Schumann | - | - | - | - | - | - | - | - | - | - | - | - | - | - | - | 3 | - | - | - | - | **3** | |
| *Staurosira construens* Ehrenberg | - | - | - | - | - | - | - | - | - | - | *-* | *-* | *-* | *-* | *-* | - | 1 | - | 2 | - | **3** | |
| *Staurosirella pinnata* (Ehrenberg) Williams e Round | - | - | - | 1 | 3 | - | - | - | - | - | *-* | *-* | *-* | *-* | *-* | 3 | - | 1 | 3 | 4 | **15** | |
| *Surirella angusta* Kützing | - | - | - | - | - | - | - | - | - | - | - | - | - | - | 2 | - | - | - | - | - | **2** | |
| *Surirella elegans*Ehrenberg | - | - | - | - | - | - | - | - | - | - | *-* | *-* | *-* | *-* | *-* | - | - | - | - | 1 | **1** | |
| *Surirella tenera* Gregory | - | - | - | 1 | 1 | - | - | - | - | - | *-* | *-* | *-* | *-* | *-* | - | - | - | 2 | 1 | **5** | |
| *Ulnaria biceps* (Kutzing) Compère | - | - | - | 1 | 2 | 14 | 21 | 32 | 9 | 38 | 57 | 32 | 64 | 43 | 83 | 2 | - | 2 | 3 | 3 | **406** | |
| *Ulnaria ulna* (Nitzsch) Compère | 1 | 4 | 1 | - | 1 | - | 14 | 4 | 5 | 4 | 19 | 18 | 31 | 11 | 19 | 1 | 2 | - | - | 2 | **137** | |
| **TOTAL** | **404** | **406** | **407** | **406** | **407** | **401** | **405** | **405** | **405** | **402** | **407** | **402** | **403** | **409** | **407** | **405** | **406** | **408** | **406** | **400** | **8101** | |

TABLE S5b. Most representative diatom species considered in the CCA calculation. Those that have fewer than 10 total frustules are excluded from the statistical calculation since they are not considered significant at the level of population structure. Each species is accompanied by the abbreviation that is then present in the CCA graph.

|  |  | ST 1 | ST 1 | ST 1 | ST 1 | ST 1 |  | ST 2 | ST 2 | ST 2 | ST 2 | ST 2 |  | ST 3 | ST 3 | ST 3 | ST 3 | ST 3 |  | ST 4 | ST 4 | ST 4 | ST 4 | ST 4 |  |  |
| --- | --- | --- | --- | --- | --- | --- | --- | --- | --- | --- | --- | --- | --- | --- | --- | --- | --- | --- | --- | --- | --- | --- | --- | --- | --- | --- |
|  | ***SPECIE OF DIATOM*** | ago-17 | may-2018 | ago-2018 | june-2019 | sept-2019 | **ST 1** | ago-17 | may-2018 | ago-2018 | june-2019 | sept-2019 | **ST 2** | ago-17 | may-2018 | ago-2018 | june-2019 | sept-2019 | **ST 3** | ago-17 | may-18 | ago-18 | june-19 | sept-19 | **ST 4** | **TOT** |
| AEJ | *Achnanthes minutissima* Kutzing var. *jackii* (Rabenhorst) Lange-Bertalot | - | - | - | 7 | 9 | **16** | - | - | - | - | - | **0** | - | - | - | 15 | 3 | **18** | - |  |  |  |  | **0** | **68** |
| AER | *Achnanthes rupestoides* Hohn | 8 | - | 7 | 2 | 2 | **19** | 3 | 2 | 3 | 2 | - | **10** | 7 | - | - | - | - | **7** | 3 | - | 5 | - | - | **8** | **80** |
| AIB | *Achnanthidium biasolettianum* (Grunow) Lange-Bertalot | - | - | 18 | - | - | **18** | - | - | - | - | - | **0** | 1 | 8 | 7 | - | - | **16** | 4 | 129 | 21 | - | 5 | **159** | **227** |
| AIL | *Achnanthidium lineare* W.Smith | 162 | 276 | 166 | 253 | 168 | **1025** | 8 | 5 | - | 25 | - | **38** | 52 | 22 | 49 | 13 | 44 | **180** | 125 | 93 | 165 | 68 | 138 | **589** | **3075** |
| AIM | *Achnanthidium minutissimum* (Kützing) Czarnecki | 20 | 6 | 13 | 8 | 49 | **96** | - | - | - | - | - | **0** | 11 | 29 | 11 | 7 | 4 | **62** | 8 | 18 | - | 89 | 44 | **159** | **475** |
| AIS | *Achnanthidium straubianum* (Langhe Bertalot) Langhe Bertalot | - | - | - | - | - | **0** | 3 | - | - | 5 | 3 | **11** | - | - | - | - | - | **0** | - | - | - | - | - | **0** | **22** |
| APP | *Amphipleura pellucida* Kützing | 5 | 8 | 2 | 1 | 6 | **22** | - | - | - | - | - | **0** | - | - | - | - | - | **0** | 8 | 2 | 16 | 5 | 13 | **44** | **88** |
| AMI | *Amphora inariensis* Krammer | 1 | 12 | 6 | 1 | - | **20** | - | 13 | - | - | - | **13** | - | 7 | - | - | - | **7** | 4 | 7 | 5 | - | 2 | **18** | **98** |
| AMO | *Amphora ovalis* (Kützing) Kützing | 1 | 3 | 2 | 5 | 6 | **17** | 5 | 3 | 2 | 2 | 1 | **13** | - | 2 | 1 | - | - | **3** | 4 | 2 | - | 3 | 1 | **10** | **76** |
| AMP | *Amphora pediculus* (Kützing) Grunow | 3 | - | - | 2 | - | **5** | 1 | - | - | 8 | - | **9** | 3 | - | - | 8 | 1 | **12** | 3 | 2 | - | - | 2 | **7** | **59** |
| CCE | *Cocconeis euglypta* Ehrenberg | - | - | 6 | - | - | **6** | - | - | 6 | - | - | **6** | - | - | - | 12 | - | **12** | - | - | 1 | - | 3 | **4** | **52** |
| CCL | *Cocconeis lineata* Ehrenberg | 11 | 8 | 11 | 36 | 21 | **87** | 3 | 2 | 11 | 7 | 24 | **47** | - | 8 | 14 | 23 | 11 | **56** | 28 | 4 | 28 | 56 | 15 | **131** | **511** |
| CCP | *Cocconeis pediculus* Ehrenberg | - | - | 4 | - | - | **4** | - | - | - | - | - | **0** | - | - | 16 | - | 6 | **22** | - |  |  |  |  | **0** | **52** |
| CCU | *Cocconeis pseudolineata* Langhe Bertalot | 7 | - | - | 1 | - | **8** | 1 | - | - | 3 | - | **4** | - | 2 | - | - | - | **2** | 8 | 3 | - | 7 | 2 | **20** | **48** |
| DAE | *Diatoma ehrenbergii* Kutzing | 1 | 2 | 6 | 1 | - | **10** | - | - | - | - | - | **0** | - | - | 1 | 2 | 3 | **6** | 1 | - | 1 | - | - | **2** | **34** |
| DIP | *Diploneis parma* Cleve | - | - | 2 | - | - | **2** | - | - | - | - | - | **0** | - | - | - | - | - | **0** | 3 | 3 | 1 | - | 3 | **10** | **14** |
| ENS | *Encyonema silesiacum* (Bleisch) Mann | - | - | 2 | - | 2 | **4** | - | 3 | 2 | 7 | - | **12** | - | 7 | 2 | 6 | 2 | **17** | - | - | 4 | 5 | 2 | **11** | **77** |
| ENM | *Encyonopsis minuta* Krammer e Reichardt | - | - | 1 | - | - | **1** | - | - | - | 5 | - | **5** | - | - | - | - | 3 | **3** | - | - | - | - | 1 | **1** | **19** |
| EUB | *Eunotia bilunaris (Ehrenberg) Schaarschmidt in Kanitz* | - | - | - | - | 1 | **1** | - | - | - | 6 | - | **6** | - | 2 | 2 | 5 | - | **9** | 1 | - | 2 | - | 1 | **4** | **36** |
| EUM | *Eunotia minor* (Kutzing) Gronow | 1 | 5 | 2 | 1 | 3 | **12** | 14 | 11 | 133 | 34 | 43 | **235** | - | 4 | 7 | 9 | 48 | **68** | - | 2 | - | - | 7 | **9** | **639** |
| FRA | *Fragilaria arcus* (Ehrenberg) Cleve | 5 | - | 11 | - | 3 | **19** | - | - | - | - | - | **0** | - | 7 | - | - | 2 | **9** | - | - | - | - | 1 | **1** | **57** |
| FRV | *Fragilaria capucina* var. *vaucheriae* (Kutzing) Lange-Bertalot | - | - | - | - | 2 | **2** | - | 2 | 1 | - | - | **3** | - | 5 | 2 | 4 | - | **11** | - | - | - | 5 | 3 | **8** | **40** |
| FRF | *Fragilaria famelica var. famelica* (Kütz.) Lange-Bert. | - | - | - | - | - | **0** | - | - | - | - | - | **0** | - | - | - | 9 | - | **9** | - | 2 | - | - | - | **2** | **20** |
| FRU | *Fragilaria ulna* var. *acus* (Kützing) Lange-Bertalot | 7 | - | - | 1 | 2 | **10** | - | - |  | - | - | **0** | 4 | - | - | 7 | 6 | **17** | 13 | - | - | 2 | 3 | **18** | **72** |
| FTV | *Frustulia vulgaris* (Thwaites) De Toni | 1 | 2 | - | - | 6 | **9** | - | 2 | 1 | - | - | **3** | - | - | 1 | - | - | **1** | 3 | 2 | 4 | - | 1 | **10** | **36** |
| GOC | *Gomphonema acuminatum* Ehrenberg | - | 2 | - | - | - | **2** | - | 1 | 1 | 2 | - | **4** | - | - | - | - | 3 | **3** | 3 | 2 | 6 | - | - | **11** | **29** |
| GOF | *Gomphonema affine* Kützing | - | - | - | - | - | **0** | - | - | - | - | - | **0** | - | - | 2 | 7 | - | **9** | - | - | 3 | - | - | **3** | **21** |
| GON | *Gomphonema angustatum* (Kützing) Rabenhorst | - | - | - | - | - | **0** | 276 | 228 | 119 | 166 | 274 | **1063** | 129 | 93 | 123 | 138 | 131 | **614** | - | - | - | 2 | 5 | **7** | **3361** |
| GOL | *Gomphonema clavatum* Ehrenberg | - | - | 7 | 3 | - | **10** | 19 | - | 1 | 4 | - | **24** | 7 | - | 17 | - | - | **24** | - | - | 5 | - | 4 | **9** | **125** |
| GOX | *Gomphonema exilissimum (*Grun) Lange-Bertalot Reichardt | - | 3 | 1 | - | 1 | **5** | - | 9 | - | - | - | **9** | - | 25 | 4 | 7 | - | **36** | 3 | - | 2 | - | - | **5** | **105** |
| GOP | *Gomphonema parvulum* (Kützing) Kützing | 5 | 3 | 2 | 2 | 1 | **13** | 7 | 6 | 7 | - | - | **20** | 28 | 79 | 44 | - | - | **151** | - | 2 | 11 | - | - | **13** | **381** |
| GOS | *Gomphonema parvulum* var. *parvulum* f. *saprophilum* Lange-Bertalot & Reichardt | - | - | - | - | - | **0** | 4 | - | - | 13 | - | **17** | 3 | - | - | 9 | - | **12** | - |  |  |  |  | **0** | **58** |
| GOT | *Gomphonema subclavatum* (Grunow) Grunow | - | 2 | - | - | - | **2** | - | 9 | - | - | - | **9** | - | 5 | - | - | 10 | **15** | - | 2 | - | - | - | **2** | **54** |
| HIA | *Hippodonta capitata* (Ehr) L-B, Metzeltin Witkowski | 1 | - | 2 | 1 | 2 | **6** | - | 1 | - | 3 | - | **4** | - | - | - | - | - | **0** | 3 | - | 3 | - | 1 | **7** | **27** |
| LUG | *Luticula goeppertiana* (Bleisch) Mann | 3 | 2 | - | - | - | **5** | - | 1 | - | - | - | **1** | - | - | - | - | - | **0** | - | - | 9 | - | - | **9** | **21** |
| MEV | *Melosira varians* Agardh | 3 | 4 | - | 3 | - | **10** | - | - | - | 7 | - | **7** | - | - | - | 13 | 4 | **17** | 3 | 6 | - | 7 | 7 | **23** | **91** |
| NAC | *Navicula cryptocephala* Kützing | 27 | 5 | 11 | 10 | 15 | **68** | 1 | 3 | 4 | 3 | - | **11** | 16 | 12 | 2 | 32 | 4 | **66** | 31 | 2 | 2 | 7 | 31 | **73** | **363** |
| NAT | *Navicula cryptotenella* Lange-Bertalot | 7 | 3 | - | - | - | **10** | - | - | - | - | - | **0** | 3 | 4 | - | - | - | **7** | 5 | 2 | - | - | - | **7** | **41** |
| NAN | *Navicula menisculus* Schumann | - | - | 4 | 1 | 3 | **8** | - | 1 | - | - | - | **1** | - | - | - | - | - | **0** | - | 4 | 2 | 5 | 1 | **12** | **30** |
| NZF | *Nitzschia fonticola* Grunow | 8 | 5 | 4 | 3 | 4 | **24** | - | 2 | 2 | - | - | **4** | 2 | - | - | - | - | **2** | - | 9 | - | 11 | 3 | **23** | **83** |
| NZG | *Nitzschia gracilis* Hantzsch | 4 | - | - | 2 | - | **6** | - | - | - | - | - | **0** | - | - | - | - | - | **0** | - | - | - | 5 | 1 | **6** | **18** |
| NZL | *Nitzschia linearis* (Agardh) W Smith | 1 | 4 | 2 | 1 | 3 | **11** | 3 | 2 | - | - | - | **5** | - | - | - | 2 | - | **2** | 1 | 6 | 3 | 5 | 10 | **25** | **61** |
| NZP | *Nitzschia palea* (Kützing) W Smith | 11 | 3 | 4 | 1 | 2 | **21** | - | 1 | - | - | - | **1** | 2 | - | - | - | - | **2** | - | - | 2 | 7 | 4 | **13** | **61** |
| NZE | *Nitzschia pellucida Grunow* | 3 | - | - | - | - | **3** | - | - | - | - | - | **0** | *-* | *-* | *-* | *-* | *-* | **0** | 7 | - | - | - | - | **7** | **13** |
| NZM | *Nitzschia perminuta (*Grunow) M.Peragallo | 4 | 7 | - | 1 | 2 | **14** | - | - | - | - | - | **0** | 2 | 2 | - | - | - | **4** | 3 | 2 | 5 | - | 3 | **13** | **49** |
| NZU | *Nitzschia pura* Hustedt | 3 | 6 | - | - | 4 | **13** | 1 | 1 | - | - | - | **2** | - | - | - | - | - | **0** | 3 | 3 | - | 4 | 6 | **16** | **46** |
| NZT | *Nitzschia tubicola*Grunow | 5 | - | - | - | - | **5** | - | - | - | - | - | **0** | 6 | - | - | - | - | **6** | - | - | - | - | 1 | **1** | **23** |
| PNM | *Pinnularia maior* (Kützing) Rabenhorst | 3 | - | - | 1 | - | **4** | - | 1 | - | 2 | - | **3** | - | 2 | - | - | - | **2** | 1 | 1 | 2 | 1 | 2 | **7** | **25** |
| PLF | *Planothidium frequentissimum(Lange-Bertalot) Round & Bukht.* | 21 | 7 | 27 | 14 | 21 | **90** | 10 | - | - | 20 | 3 | **33** | 9 | - | - | 1 | - | **10** | 22 | 11 | 3 | 14 | 2 | **52** | **318** |
| PLL | *Planothidium lanceolatum* (Kütz ex Bréb) L-B | 10 | 2 | 10 | 2 | 8 | **32** | 12 | 43 | 63 | 46 | 9 | **173** | 18 | - | - | 2 | 12 | **32** | - | - | 2 | 6 | 1 | **9** | **483** |
| PLR | *Planothidium rostratum* (Oestrup) Lange-Bertalot | 16 | 3 | 20 | 2 | 13 | **54** | 7 | 6 | - | - | - | **13** | - | - | - | 2 | - | **2** | 51 | 15 | 41 | 32 | 29 | **168** | **306** |
| PSO | *Psammothidium oblongellum*(Oestrup) Van de Vijver | - | - | 4 | 1 | - | **5** | 3 | - | - | 5 | - | **8** | - | - | - | - | - | **0** | 1 |  | 2 |  |  | **3** | **29** |
| RES | *Reimeria sinuata* (Gregory) Kociolek Stoermer | 3 | - | 12 | 23 | 3 | **41** | - | - | 7 | - | - | **7** | - | - | - | - | - | **0** | - | 3 | 4 | - | 8 | **15** | **111** |
| RHA | *Rhoicosphenia abbreviata* (Agardh) Lange-Bertalot | 5 | 5 | 2 | 2 | 6 | **20** | 1 | 2 | 2 | - | - | **5** | *-* | *-* | *-* | *-* | *-* | **0** | 3 | 42 | 24 | 17 | 7 | **93** | **143** |
| SEP | *Sellaphora pupula* (Kützing) Mereschkowsky | 4 | 3 | 2 | - | 1 | **10** | - | 1 | - | 4 | - | **5** | 9 | 4 | - | - | - | **13** | 4 | 3 | 2 | 5 | 1 | **15** | **71** |
| STA | *Stauroneis anceps* Ehrenberg | 3 | 7 | - | - | - | **10** | 3 | 1 | - | - | - | **4** | - | - | - | - | - | **0** | - | 6 | - | - | - | **6** | **34** |
| STK | *Stauroneis kriegeri* R.M.Patrick | - | - | - | - | - | **0** | - | - | - | - | - | **0** | - | 12 | - | - | - | **12** | - |  |  |  |  | **0** | **24** |
| SSP | *Staurosirella pinnata* (Ehrenberg) Williams e Round | - | - | - | 1 | 3 | **4** | - | - | - | - | - | **0** | *-* | *-* | *-* | *-* | *-* | **0** | 3 | - | 1 | 3 | 4 | **11** | **19** |
| ULB | *Ulnaria biceps* (Kutzing) Compère | - | - | - | 1 | 2 | **3** | 14 | 21 | 32 | 9 | 38 | **114** | 57 | 32 | 64 | 43 | 83 | **279** | 2 | - | 2 | 3 | 3 | **10** | **802** |
| ULU | *Ulnaria ulna* (Nitzsch) Compère | 1 | 4 | 1 | - | 1 | **7** | - | 14 | 4 | 5 | 4 | **27** | 19 | 18 | 31 | 11 | 19 | **98** | 1 | 2 | - | - | 2 | **5** | **269** |

TABLE S6. Abiotic and Biotic biodegradation of *o-, m-, p*- xylene and total petroleum hydrocarbons C ≤ 12 and C>12 in slurry soils experiment.

| Samples | ***o-, m-, p*- xylene** | | **C ≤ 12** | | **C > 12** | |
| --- | --- | --- | --- | --- | --- | --- |
|  | Initial concentration (mg kg^-1^ s.s.) | Final concentration (mg kg^-1^ s.s.) | Initial concentration (mg kg^-1^ s.s.) | Final concentration (mg kg^-1^ s.s.) | Initial concentration (mg kg^-1^ s.s.) | Final concentration (mg kg^-1^ s.s.) |
| **SB1** | 0.885±0.009 | 0.276±0.006 | 237±71 | 155±23.5 | 962±80 | 950±81 |
| **SB1 Abiotic** | 0.303±0.034 | 0.274±0.005 | 11±1.2 | 11±1.2 | 985±111.4 | 980±102 |
| **SB2** | 0.376±0.04 | 0.274±0.022 | 6.7±1.2 | 6±0.9 | 41±0.7 | 18±3.5 |
| **SB2 Abiotic** | 0.196±0.005 | 0.197±0.006 | bdl | bdl | bdl | bdl |
| **SA** | 0.621±0.057 | 0.450±0.021 | 174±15 | 87±9 | 20459±297 | 20162±4119 |
| **SA Abiotic** | 0.680±0.096 | 0.560±0.064 | 173±6.7 | 172±4.5 | 19577±5870.6 | 19577±5027.3 |
| **SB3** | 0.305±0.018 | 0.276±0.015 | 25±7 | 9±3 | 207±8 | 141±6.1 |
| **SB3 Abiotic** | 0.301±0.001 | 0.288±0.001 | bdl | bdl | 176±9.9 | 156±7.7 |

bdl*, below detection limit*

TABLE S7. Identification of the selected strains and results obtained on PGP tests, growth and adhesion on Hexadecane (C16) and Zn(II) resistance.

| OTU | Sample | Accession number | Species | Growth on C16 | Adhesion to C16 | N2 Fixation | P solubilization | IAA production | Siderophore production | EPS production | Motility | Zn(II) resistance |
| --- | --- | --- | --- | --- | --- | --- | --- | --- | --- | --- | --- | --- |
| 1 | SB1-17 | PP272392 | *Bacillus mobilis* | - | + | - | - | - | - | - | - | ND |
| 2 | SA-13 | PP272401 | *Bacillus mycoides* | - | - | - | - | - | - | - | - | ND |
| 3 | SB3-12 | PP272396 | *Bacillus asahii* | - | + | - | - | - | - | - | - | ND |
| 4 | SA-2 | PP272398 | *Bacillus mobilis* | - | + | - | - | - | + | - | + | ND |
| 5 | BULK-MG8 | PP272407 | *Viridibacillus arenosi* | - | + | - | - | + | - | - | + | ND |
| 6 | SB1-13 | PP272391 | *Paenibacillus glucanolyticus* | - | + | - | - | - | - | - | - | ND |
| 7 | SB1-21 | PP272393 | *Planomicrobium glaciei* | - | + | - | - | - | - | - | - | ND |
| 8 | SB3-2 | PP272394 | *Acinetobacter calcoaceticus* | + | + | - | - | - | - | - | - | ND |
| 9 | BULK-LC6 | PP272406 | *Brucella thiophenivorans* | - | - | - | - | + | - | + | + | ND |
| 10 | SB3-4 | PP272395 | *Micrococcus luteus* | - | + | - | - | - | - | - | - | ND |
| 11 | SB3-16 | PP272397 | *Micrococcus luteus* | - | - | - | - | - | - | - | - | ND |
| 12 | SA-3 | PP272399 | *Brachybacterium paraconglomeratum* | - | - | - | - | - | - | - | - | ND |
| 13 | SA-18 | PP272402 | *Comamonas koreensis* | - | + | - | - | + | - | - | - | ND |
| 14 | SA-12 | PP272400 | *Rhodococcus kronopolitis* | + | + | + | - | + | - | - | - | ND |
| 15 | BULK-LC2.2 | PP272405 | *Rhodococcus pedocola* | + | - | - | - | - | - | - | - | ND |
| 16 | RZ-AC8 yellow | PP272403 | *Streptomyces fulvissimus* | - | + | + | - | + | + | - | - | ND |
| 17 | RZ-AC8 red | PP272408 | *Streptomyces californicus* | - | - | + | - | - | + | - | + | ND |
| 18 | BULK-LC1.2 | PP272404 | *Rhodococcus qingshengii* | + | + | - | - | + | - | + | + | ND |
| 19 | MORI-15 | PP272410 | *Pseudomonas koreensis* | - | - | - | - | + | + | - | + | - |
| 20 | MORI-38 | PP272409 | *Pseudomonas koreensis* | - | - | + | - | + | + | - | + | - |
| 21 | MORI-53 | PP272411 | *Aeromonas media* | - | + | + | - | + | + | - | + | + |

*ND., not determined*

TABLE S8. Roots, shoots and total biomass dry weight (105°C until constant weight) of *Zea mays* non-inoculated and inoculated with *Aeromonas media* strain MORI-53, *Pseudomonas koreensis* MORI-15 and *Rhodococcus kronopolitis* SA-12.

|  | Roots mg d.w. | Shoots mg d.w. | Total biomass mg d.w. |
| --- | --- | --- | --- |
| Uninoculated control | 20±2.4 | 20±1.90 | 40±1.63 |
| *Aeromonas media* strain MORI-53 | 30±1.3 | 35±2.34 | 65±2.28 |
| *Pseudomonas koreensis* strain MORI-15 | 30±1.14 | 10±2.34 | 40±2.13 |
| *Rhodococcus kronopolitis* strain SA-12 | 15±2.46 | 17.5±1.5 | 32.5±3.13 |

FIGURE S1. Shoot bioconcentration factor of C>12 hydrocarbons in *Zea mays* and *Helianthus annus* grown in SB3 and WT soil mesocosms, after 38 days.


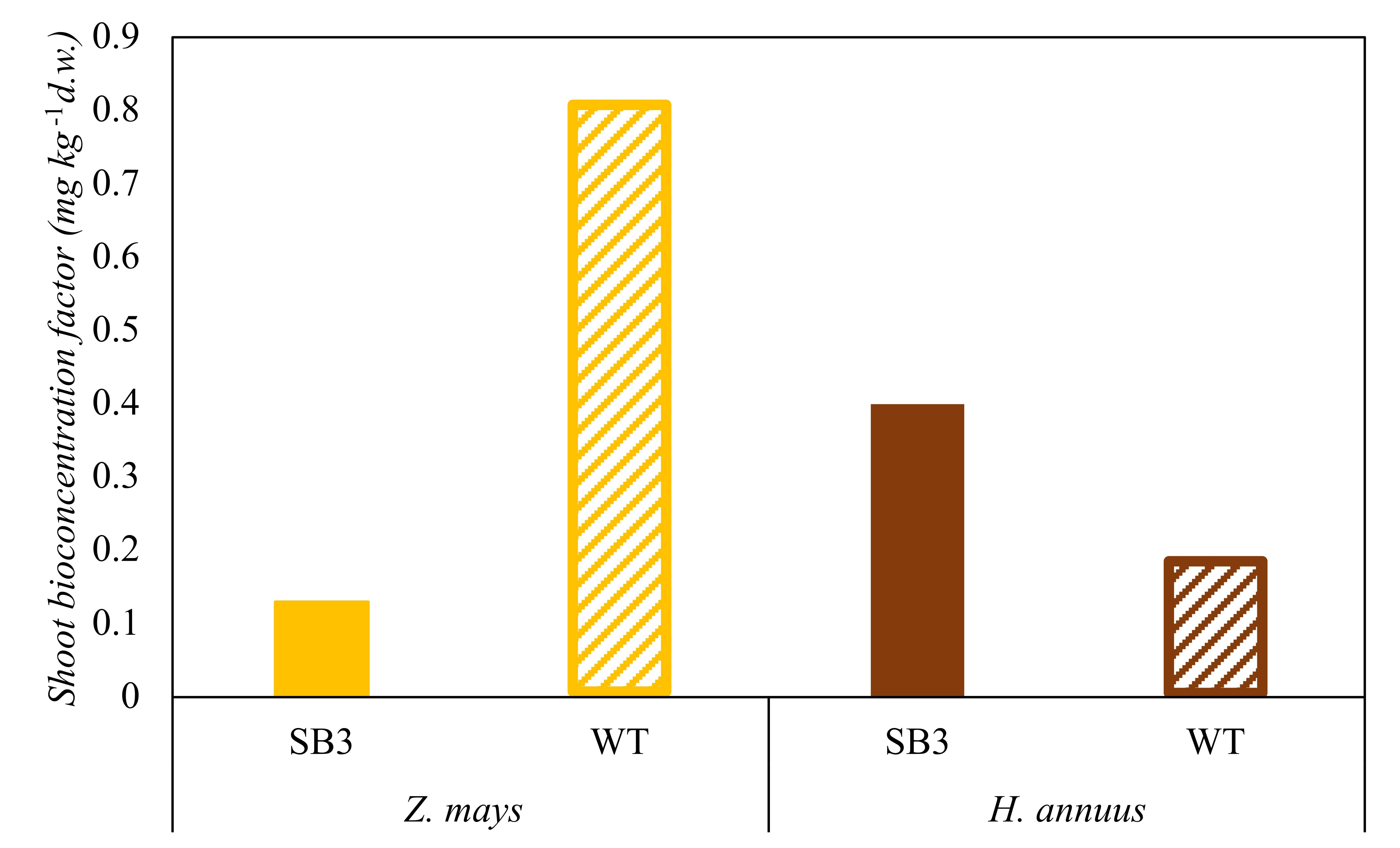

Supplement: Supplementary file 1 [file DataSheet1.docx]
